# Synthesis of Monodisperse PbS/CdS Colloidal Quantum Dots Emitting at Telecommunication Wavelengths with suppressed Auger Rates and Gain Threshold

**DOI:** 10.1021/acs.nanolett.6c00158

**Published:** 2026-02-26

**Authors:** Marios Stylianou, Eric G. Bowes, Luca Leoncino, Rosaria Brescia, Victoria Nisoli, Nathan A. Malone, Jessica Q. Geisenhoff, Jennifer A. Hollingsworth, Andreas Othonos, Sotirios Christodoulou

**Affiliations:** † Inorganic Nanocrystals Laboratory, Department of Chemistry, 54557 University of Cyprus, Nicosia 1678, Cyprus; ‡ Materials Physics & Applications Division: Center for Integrated Nanotechnologies, 630239 Los Alamos National Laboratory, Los Alamos, New Mexico 87545, United States; § Electron Microscopy Facility, Instituto Italiano di Tecnologia, Genova 16163, Italy; ∥ Laboratory of Ultrafast Science, Department of Physics, University of Cyprus, Nicosia 2109, Cyprus

**Keywords:** PbS/CdS CQDs, Auger rates, optical gain, telecommunication wavelengths

## Abstract

Semiconductor nanomaterials that combine high near-infrared (NIR) photoluminescence efficiency and photostability are limited. Pb-chalcogenide colloidal quantum dots (CQDs), and particularly PbS CQDs, are promising candidates. Nevertheless, the practical implementation of PbS CQDs in optical devices faces intrinsic limitations due to the 8-fold degeneracy of the conduction and valence bands, leading to enhanced nonradiative Auger recombination, hindering applications such as lasing. Here, we focus on the synthesis and optical characterization of core–shell PbS/CdS CQDs emitting at telecommunication wavelengths (1500–1620 nm) with suppressed Auger rates. We synthesized three series of core/shell PbS/CdS CQDs from different-sized PbS cores via a cation exchange reaction. This approach produces PbS/CdS CQDs with high optical stability and narrow size distribution. Finally, we optically probed the nanocrystals with transient absorption, demonstrating suppressed Auger rates, increasing biexciton Auger lifetimes τ_*xx*_ up to 320 ps, while reducing the gain threshold of the system down to ⟨*N*⟩_gain_≈ 1.7.

## Graphical abstract

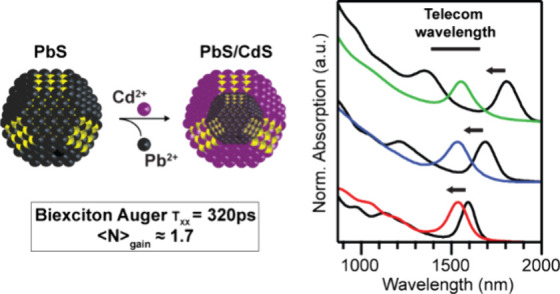

Among the plethora of nanoscale materials, colloidal quantum dots (CQDs) have emerged as a class of nanocrystals (NCs) with great potential due to their excellent optoelectronic properties such as tunable bandgap, enhanced photoluminescence quantum yield (PLQY) and high optical stability.^1–7^ Therefore, in the visible regime, CQDs have already been employed successfully in optoelectronic devices.^8,9^

Nevertheless, due to the rapid advancement of near-infrared (NIR) technologies including versatile applications such as optical sensing, imaging and telecommunications, the demand for materials capable of emitting in the lower energy range of the electromagnetic spectrum has become imperative.^10,11^ A promising NIR semiconductor is lead­(II) sulfide (PbS). In particular, PbS CQDs can provide a high degree of spectral tunability (600–2600 nm).^12,13^ PbS CQDs have been successfully employed in NIR optoelectronics, such as LEDs,^14^ solar cells^15^ and photodetectors^16,17^ showing state-of-the art performance. Nevertheless, the existence of high degeneracy (8-fold) in the conduction and valence band in PbS CQDs increases the multiexciton interaction, resulting in enhanced Auger recombination rates, and combined with the ease of surface oxidation constitutes an important drawback for the realization of stable low-threshold lasing operating in ambient conditions.

Biexciton Auger lifetimes τ_*xx*_ from dynamic studies on Pb-chalcogenide systems such as PbS­(Se) range from 38 ps up to 160 ps, depending on the diameter of the core material.^18–21^ To suppress the high rates of nonradiative Auger recombination, various doping techniques^22–25^ and use of permanently charged CQDs^26,27^ have been employed. In heavily n-doped PbS CQDs, for instance, chemical doping combined with ligand exchange using 1-ethyl-3-methylimidazolium iodide (EMII) has led to band-edge filling that supports stimulated emission from single excitons, reducing optical gain thresholds from ∼4 excitons per dot in undoped systems to ⟨*N*⟩ ≈ 1.^22^ This enabled direct observation of single-exciton gain in doped PbS CQDs, a major milestone for CQD optoelectronics.^22^ Nevertheless, the doping requires prohibitively expensive epitaxial growth of Al_2_O_3_, via atomic layer deposition methods, as ligand-exchange QD-solids do not show any permanent doping.

Core/shell heterostructuring has also been used to influence Auger processes, with the shell providing further advantages, from enhanced photoluminescence efficiencies to improved stability. The encapsulation of the core material within a shell passivates the surface,^28^ reducing surface defects (trap states). In addition, for some core sizes^29^ or ultrathin shells shelling can allow delocalization of a carrier’s (electron or hole) wave function into the shell, forming a quasi-type II band alignment reducing the Auger rates. A recent study reported the suppression of Auger recombination in PbS/PbSSe alloyed core/shell CQDs, achieving biexciton Auger lifetimes up to 485 ps.^30^ In this case interfacial alloying caused both electron and hole wave functions to penetrate into the shell and carrier spatial overlap density function to span the whole of the nanocrystal, contributing to Auger recombination suppression, combined with chemical doping and epitaxial growth of Al_2_O_3_ for further stabilization, optical gain at sub-single-exciton occupancies with ASE thresholds as low as ⟨*N* ⟩ ≈ 0.7 have been achieved.^30^ In addition, alloying smoothens interfacial potential energy confinement, which can also reduce Auger recombination.^31^

PbS/CdS core/shell CQDs are synthesized by different techniques, such as cation exchange,^32,33^ c-ALD^34,35^ or a 2-step procedure employing cation exchange followed by successive ionic layer adsorption and reaction (SILAR)^36^ to precisely control the thickness of the CdS shell on the nanometer scale. The choice of the shell-growth method can impact optical performance. For example, the PLQY of PbS/CdS CQDs prepared by the c-ALD approach is reduced in comparison to PbS cores due to the presence of defects at the interface between PbS and CdS.^35^

Multiexciton Auger dynamics in PbS/CdS CQDs have been investigated via transient absorption (TA), determining instantaneous and delayed generation processes of multiple excitons^37^ showing a fast decay within the first 200 ps that corresponds to Auger recombination of multiple excitons.^37,38^ Biexciton Auger lifetimes that have been obtained were equal to 47 ps^37^ and ∼60 ps,^38^ for an average PbS core diameter of 5.2 and 4.6 nm, respectively. Moderate improvements have also been made successfully for PbS/CdS core/shells emitting at 950 and 1300 nm, achieving τ_*xx*_ = 35 ± 8 ps and τ_*xx*_ = 126 ± 10 ps,^29^ respectively.

Limited studies on photostable PbS/CdS CQDs emitting in the O- (1260–1360 nm) and S- (1460–1530 nm) telecom bands have been reported,^36^ while their optical response under multiexcitonic regime still remains elusive. However, achieving gain thresholds that approach the subexciton regime solely through heterostructuring, comparable with CdSe/CdS systems, without the use of external doping techniques^22–25^ and permanently charged CQDs,^26,27^ is still widely unexplored, while only PbS-based systems with post-encapsulation approaches have been explored. Here, we show an extensive study of PbS/CdS CQDs series, varying both the core and the shell size, targeting further into the telecommunication wavelengths to include the C- and L-bands (1530 nm–1625 nm). We synthesized three series of core/shell PbS/CdS CQDs from different-sized PbS cores with suppressed Auger rates and tunable band-edge absorption across the telecommunication wavelength bands. The epitaxial growth of the CdS shell was achieved via controlled cation exchange reaction, producing CQDs of high optical stability and narrow size distribution. Despite the reduction of PbS core size after the shell formation via cation exchange, we observed significantly extended Auger lifetimes of up to 320 ps. Moreover, we significantly reduced the optical gain threshold, reaching a value of ⟨*N* ⟩_gain_ ≈ 1.7, which approaches the single-exciton regime and represents a substantial reduction compared to the four-exciton threshold of PbS CQDs, and high optical stability without the need for an epitaxial encapsulation. This was achieved solely by precisely forming heterostructures via the hot-injection synthesis method.

The synthesis of PbS CQDs follows the procedure developed by Hines et al.^39^ using lead­(II) oleate [Pb­(oleate)_2_] and hexamethyldisilathiane [(TMS)_2_S] as the Pb^2+^ and S^2–^ precursors, respectively (Figure 1a). To achieve the synthesis of target-sized CQDs with narrow size distribution and sharp optical features, we optimized the reaction’s temperature and the concentration of S-precursor, managing to control the nucleation and growth steps of the reaction. Therefore, utilizing high concentrations of S-precursor (from ∼0.5 to 1.5 M) at high injection temperatures (θ = 115 °C) while carrying out multiple injections, we achieved monodispersed colloidal dispersions of three different sized PbS CQDs with absorption at ∼1600, ∼1700, and ∼1800 nm, respectively, exhibiting high crystallinity and a narrow size distribution of ∼4–6% (Table 1, Supporting Information - Experimental section, S1–3)

**Figure 1:**
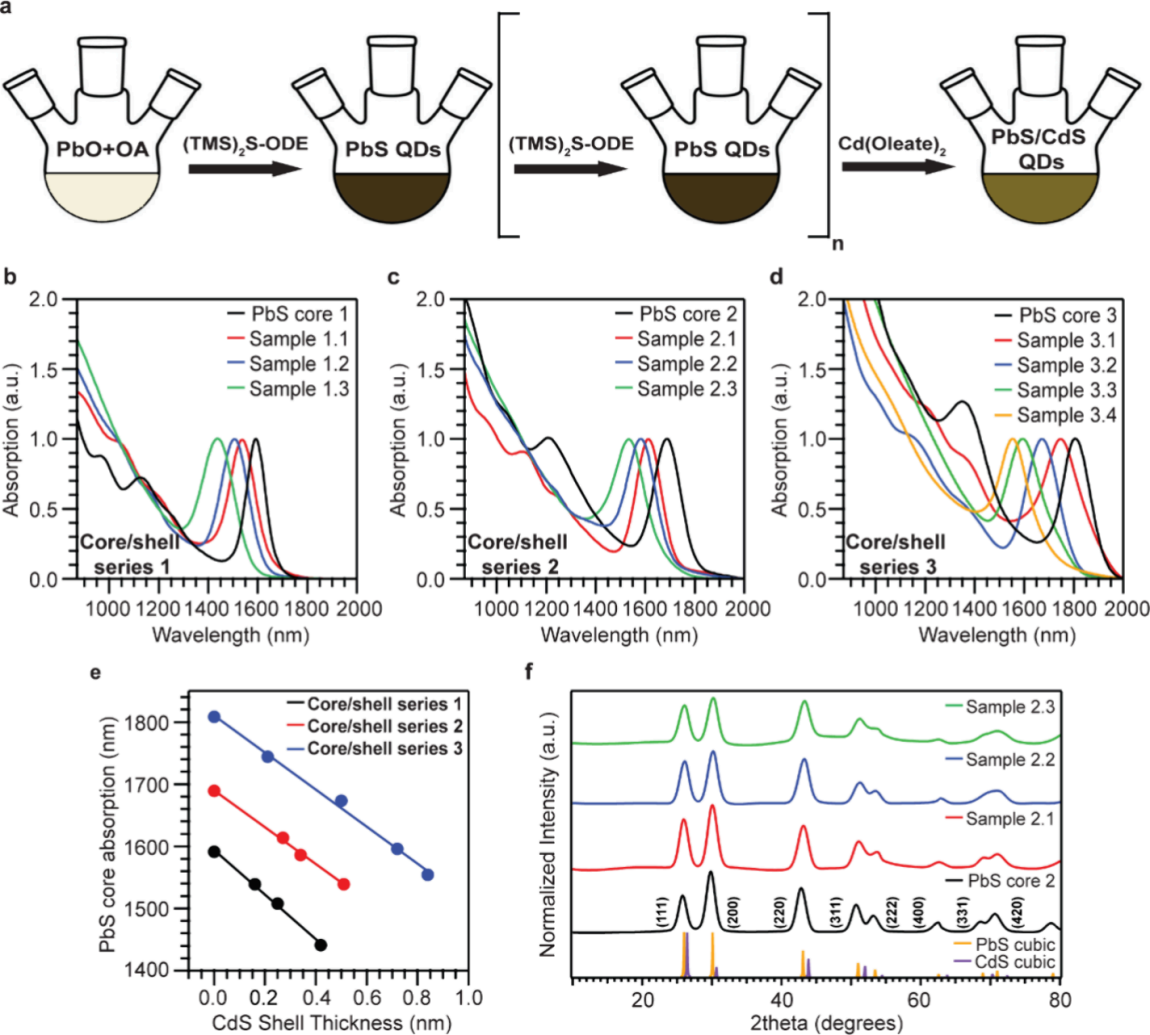
(a) Scheme of PbS CQDs and PbS/CdS core/shell synthesis. (b, c, d) UV-NIR spectra of core/shell series 1, 2, and 3, respectively. (e) PbS core absorption versus CdS shell thickness in the three core/shell series. (f) Powder X-ray diffraction (pXRD) spectra of core/shell series 2. Theoretical patterns of cubic rock-salt PbS (COD No. 5000087) and cubic zinc blende CdS (COD No. 9008839).

**Table 1:** Cation Exchanged PbS/CdS Core/Shell Series^a^

PbS/CdS core/shell series		Reaction time (min/h)	Exciton absorption (nm)	fwhm/σd (nm/%)	PbS core diameter (nm)	CdS shell thickness(nm)
Series 1	PbS core 1	-	1591	92.53/3.84	6.18	-
	Sample 1.1	2	1539	111.06/4.65	5.87	0.16
	Sample 1.2	10	1508	128.97/5.42	5.69	0.25
	Sample 1.3	40	1441	146.86/6.26	5.32	0.42
Series 2	PbS core 2	-	1689	123.57/5.11	6.79	-
	Sample 2.1	2	1614	118.22/4.91	6.32	0.27
	Sample 2.2	40	1586	127.05/5.28	6.15	0.34
	Sample 2.3	160	1539	155.28/6.50	5.87	0.51
Series 3	PbS core 3	-	1808	130.13/5.41	7.63	-
	Sample 3.1	40	1744	273.65/11.33	7.17	0.21
	Sample 3.2	160	1673	152.53/6.31	6.69	0.50
	Sample 3.3	13 h 40 min	1596	144.47/6.00	6.21	0.72
	Sample 3.4	50 h	1554	137.16/5.73	5.96	0.84

a Reaction time and observed excitonic absorption were obtained from UV-NIR spectra. Full width half maximum (FWHM), size distribution σd, PbS core diameter. and CdS shell thickness from Maxwell–Garnett analysis.^40^

For the PbS/CdS core/shell CQDs, we employed a similar procedure adapted by Pietryga et al.^41^ We started from the three initial PbS cores and injected a stock solution of 0.3 M cadmium­(II) oleate [Cd­(oleate)_2_] for the cation exchange reaction to take place and we varied the reaction’s time (from 2 min to 50 h) fabricating CdS shells of different thickness (Table 1, Supporting Information—Experimental section). The schematic of the reaction is illustrated in Figure 1a. The optical properties and the cation exchange reactions on the three different-sized PbS CQDs have been probed with absorption spectra showing a blue shift of the exciton peak as the CdS shell increases simultaneously with the reduction of PbS core size (Figure 1b, c, d). As a result, we achieved the tuning of the band-edge transition of the PbS core material in the three-cation exchanged PbS/CdS core/shell series, reaching down to the optimal telecommunication window (∼1550 nm; Table 1). The high quality of colloidal PbS/CdS is translated in sharp optical absorption features at high energies (1S­(h)–1S­(e), 1P­(h)–1P­(e), 1D­(h)–1D­(e)) which is an indication that despite the cation exchange reactions at high temperature, the crystallinity of the CQDs remains intact, and also the narrow size distribution is maintained (Table 1, Supporting Information S1–3). In contrast, previous results have shown significant loss in monodispersity with extended cation exchange.^32,42–44^

First, we studied the influence of the core size retaining the shell thickness the same. This is not trivial, as we carefully synthesized three core sizes from ∼6.2 to ∼7.6 nm, which later we kinetically controlled their cation exchange reaction achieving a CdS shell thickness of ∼0.5 nm (Table 1—Samples 1.3, 2.3, 3.2). The reaction time for the synthesis of ∼0.5 nm shell varied based on the initial core size. Therefore, for the PbS core of core/shell series 1 the cation exchange reaction was 40 min and for the PbS cores of the core/shell series 2 and 3, was 160 min, respectively. Despite the size difference of the larger cores, PbS core 2 and 3 (approximately 1 nm difference in size), the fabrication of the same CdS shell thickness needed the same reaction time. This is an indication that the diffusion rate is comparable in cation exchange reactions on large cores (∼>7 nm) and therefore variations in core size do not significantly influence the reaction.

However, in order to tune gradually the band-edge transition of the PbS core 3 down to the telecom band fabricating a thick CdS shell, the synthesis of Sample 3.3 required a second injection of [Cd­(oleate)_2_] at 3 h of cation exchange reaction whereas the synthesis of Sample 3.4 required a second and a third injection at 3 h and approximately 28 h of reaction time, respectively (Supporting Information S4). In both cases, the CdS shell growth slows down logarithmically. After the first injection, the process kinetically approaches a plateau after approximately 3 h of reaction, resulting in the formation of a ∼0.5 nm of CdS shell. This suggests that the cation reaction reaches a precursor limit, beyond which further shell formation is not feasible without the additional introduction of the Cd-precursor. We observed similar growth of the CdS shell after the second injection. In contrast to the first injection, after approximately 24 h of cation exchange reaction, the system does not reach a kinetic plateau. However, in order to enhance the reaction kinetics by reducing the time needed to achieve a CdS shell thickness sufficient to tune the band-edge transition of PbS core 3 down to the desired telecom wavelength, we performed a third injection of the Cd-precursor. The third injection also follows logarithmic growth, leading to the synthesis of Sample 3.4 after 50 h of cation exchange reaction. Figure 1e summarizes the band-edge absorption wavelength of the PbS core in each cation-exchanged PbS/CdS CQD in relation to the CdS shell thickness. We can observe a blue shift of the exciton’s absorption wavelength as the CdS shell thickness increases in all three core/shell series, indicating a linear relation between bandgap transition of PbS core and the thickness of CdS shell. Specifically, the reduction of PbS core diameter from ∼6.2 to ∼5.3 nm in core/shell series 1, from ∼6.8 to ∼5.9 nm in core/shell series 2 and from ∼7.6 to ∼5.9 nm in core/shell series 3 has led to a successful blue shift of absorption (Figure 1e, Table 1) and thereby in photoluminescence (Supporting Information S5) reaching the telecommunication wavelength range.

To probe the CQD crystal structure, we performed powder X-ray diffraction (pXRD) (Figure 1f, Supporting Information S1). All the core and core/shell pXRD patterns present high crystallinity, following a cubic crystal structure. In Figure 1f, we show the pXRD pattern of core/shell series 2, of both the initial PbS core and the PbS/CdS core/shell samples. With a closer analysis of the pXRD patterns (Supporting Information Table S1), we observed a slight red shift in the diffraction peaks as we grew a thicker CdS shell. This is attributed to the dominance of CdS in the PbS/CdS nanoparticle’s volume since the cation exchange reaction proceeds with the formation of the shell and the reduction of core size. In addition, as encapsulation proceeds, some diffraction peaks are broadened or have shoulders due to the coexistence of peaks for the two lattice phases, rock-salt of PbS, and zinc blende of CdS. The ratio of the peak intensities corresponding to (111) and (200) crystal planes is also reduced as the shell thickness increases.

Local analysis of the crystal structure of PbS/CdS core/shell CQD performed via high-resolution transmission electron microscopy (HRTEM), confirms the successful formation of the heterostructure, which consists of a ∼4.5 nm core and a ∼0.85 nm thick shell in size, respectively, for Sample 3.3 (Figure 2a). We further notice that the core/shell interface is not very defined; therefore, by combining the particle and the shell size from the absorption data which indicates a slightly bigger dot and thinner shell, we may conclude that we have an alloyed Pb_*x*_Cd_*x*–1_S interface, while the exciton in the core will be delocalized more efficiently in the interface. Performing peak pair analysis (PPA) of the image, we obtained the mean dilation map which indicates that the cell parameters in the core are about 2.3% larger than in the shell (Figure 2b). Therefore, we expect that due to the mild conditions utilized for the cation exchange reaction, we achieved an epitaxial growth of the shell and the creation of an alloyed interface, which is supported with the PPA, showing a dilation at the core/shell interface. In addition, Fast Fourier Transformation (FFT) of the image in selected regions in the core/shell CQD (red frame for the core and green frame for the shell in Figure 2a) shows a [211]-oriented cubic structure (Figure 2c, d). Finally, high-angle annular dark-field scanning transmission electron microscopy (HAADF-STEM) imaging was performed on three PbS/CdS CQDs. The images reveal a slight intensity contrast, with the central region of the nanoparticles exhibiting higher intensity relative to the periphery. This brightness enhancement is attributed to the higher mean atomic number within the PbS core (Figure 2e). To further investigate the compositional distribution, elemental line profiles were extracted from scanning transmission electron microscopy-energy dispersive X-ray spectroscopy (STEM-EDS) maps across the three nanoparticle centers (Figure 2f). The profiles show alternating peaks and dips in the Pb and Cd signals, consistent with core–shell structures and their intermediate regions, with high overlap over the Pb and Cd signal beyond the core region, which further supports the creation of an alloyed interface. Particularly, the central region contains overlapping signals from both Pb and Cd, whereas the outer edges are enriched in Cd-atoms, confirming the formation of a CdS shell encapsulating a PbS-rich core. The observed elemental distribution corroborates the contrast observed in HAADF-STEM images supporting the structural integrity of the core/shell heterostructure.

**Figure 2:**
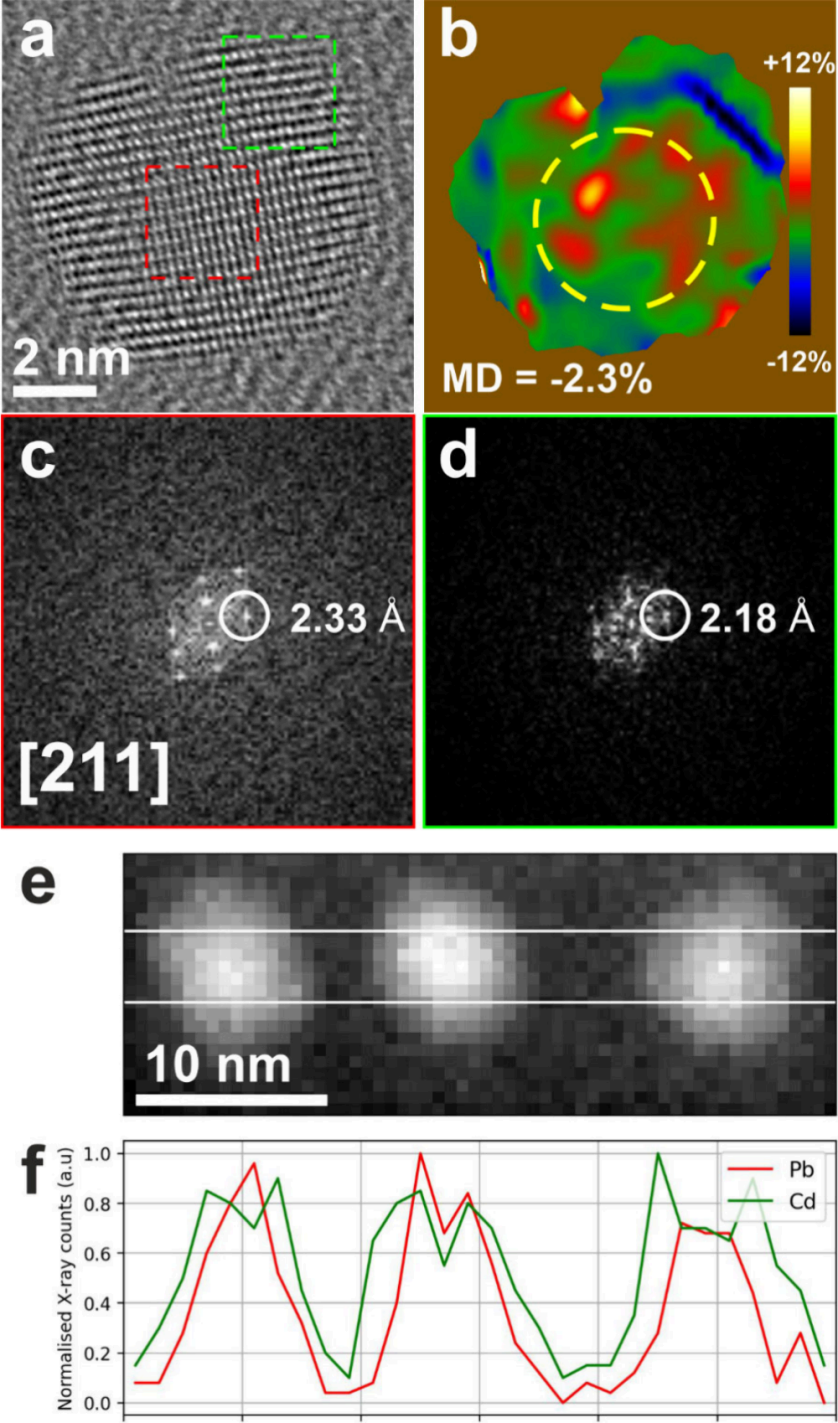
(a) HRTEM image of PbS/CdS CQD (Sample 3.3) and (b) corresponding mean dilation map calculated by PPA. The cell parameters in the outer region of the particle are about 2.3% smaller than those in the core area (within the circled area). (c) and (d) are fast Fourier transforms (FFTs) of areas framed in (a): both regions show a [211]-oriented cubic structure. The one in (c) closer to the PbS structure (ICSD 38293), the one in (d) to sphalerite CdS (ICSD 81925). (e) HAADF-STEM image of three nanoparticles and (f) line profiles computed from STEM-EDS maps, showing the variation of Pb and Cd content along the central region of three nanoparticles (framed in panel (e)).

Delving into the multiexcitonic dynamics of our CQDs, we performed transient absorption (TA) measurements probing the Auger recombination. To this end, we fabricated conductive CQD-films of ∼170 nm thickness via layer-by-layer deposition. The CQD-films were fabricated in line with the literature using a combination of two ligands, 1-ethyl-3-methylimidazolium (EMII) and 3-mercaptopropionic acid (MPA). The TA measurements were carried out employing a Ti/Sapphire femtosecond laser pumping the samples at 800 nm. We studied the Auger dynamics in all the core and core/shell samples at exciton occupancy ⟨*N*⟩ ≈ 0.4 (Figure 3a-c). The exciton occupancy has been determined considering a close pack film of cuboctahedra shaped CQDs, resulting in a packing density of φ_L_max = 0.92 ^45^ (Supporting Information S6), while the TA traces have been fitted with biexponential decay, probing both the single and biexciton relaxations.

**Figure 3:**
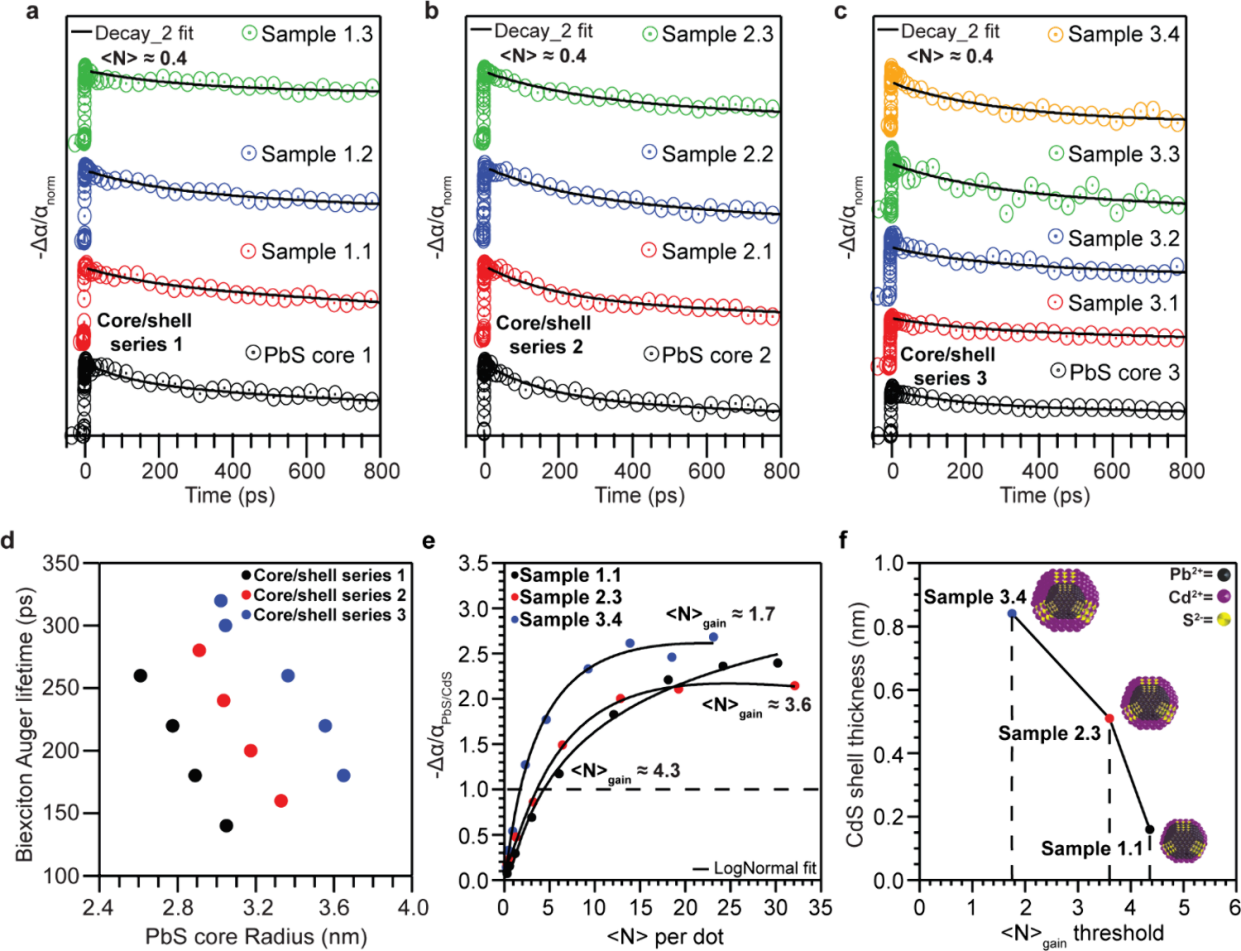
(a, b, c) Transient absorption (TA) curves of PbS/CdS samples of each core/shell series at exciton occupancy ⟨*N*⟩ ≈ 0.4 where the carrier dynamics are dominated by Auger recombination. d) Calculated biexciton Auger lifetimes of the core/shell series using a double exponential decay fit on the TA curves, showing their correlation with PbS core radius. Gain spectra have been obtained in probing wavelengths, 50 nm red-shifted from the band-edge absorption of the materials reaching the stimulated emission regime and using different pump fluences (0.5–50 μJ/pulse) which are presented as exciton occupancy ⟨*N*⟩ per dot. The optical gain arises at the point where -Δα/α_PbS/CdS_ > 1 (horizontal black dash line). e) Gain spectra of three PbS/CdS CQDs samples with the same PbS core of ∼5.9 nm (exciton peak at ∼1550 nm—optimal telecom wavelength) and different CdS shell thickness. f) Observed ⟨*N*⟩_gain_ values in relation to CdS shell thickness in Samples 1.1, 2.3, and 3.4, respectively.

In all core–shell series, we show a linear relation of the CdS shell thickness to Auger rates. In fact, the TA traces show an enhancement of the τ_*xx*_ in the thicker shell, due to the creation of the alloyed interface, which is more pronounced in CQDs with a thicker shell.^46,47^ We have to notice that due to the cation exchange reaction of the PbS/CdS, the size of the CQDs remains the same; thereby, the total QD volume is intact. Nevertheless, in all series we observed that increasing the CdS shell thickness and consequently reducing the core volume reduced the Auger lifetimes were suppressed. Specifically, τ_*xx*_ was increased from 140 to 260 ps, 160 to 280 ps and 180 to 320 ps for PbS/CdS core/shell series 1, 2 and 3, respectively (Table 2). We observed that CQDs from the core/shell series 3, which originated from the largest PbS core 3, exhibit increased Auger lifetimes compared with the other series. This is attributed to the larger total exciton volume of the initial PbS core 3 and to the alloyed interface.

**Table 2:** Biexciton Auger Lifetimes τ*_xx_* of PbS/CdS Core/Shell Series Extracted from Double Exponential Decay Fit on the TA Traces at ⟨*N*⟩ ≈ 0.4

PbS/CdS core/shell series		Biexciton Auger lifetime τ_*xx*_ (ps)
Series 1	PbS core 1	140
	Sample 1.1	180
	Sample 1.2	220
	Sample 1.3	260
Series 2	PbS core 2	160
	Sample 2.1	200
	Sample 2.2	240
	Sample 2.3	280
Series 3	PbS core 3	180
	Sample 3.1	220
	Sample 3.2	260
	Sample 3.3	300
	Sample 3.4	320

Figure 3d summarizes the biexciton Auger lifetimes τ_*xx*_ of all core and core/shell CQDs. Within individual core/shell series, CQDs with comparable PbS core sizes but varying CdS shell thicknesses such as PbS core 1, Sample 2.2 and Sample 3.3 (PbS core size ∼6.2 nm, CdS shell thickness ∼0.35 nm and ∼0.7 nm for Sample 2.2 and Sample 3.3, respectively) exhibit distinguishable biexciton Auger lifetimes, with the thickest shelled heterostructure demonstrating the most significant suppression of Auger recombination. This outcome validates the efficacy of the cation exchange-based shelling strategy and underscores the critical role of shell thickness and the importance of the alloyed interface reducing nonradiative decay pathways. This is further supported by direct comparison between bare PbS core 3 and core/shell Sample 1.1. Due to its origin from the smaller PbS core 1, Sample 1.1 exhibits a smaller total exciton volume compared to the larger PbS core 3. However, the presence of a CdS shell in Sample 1.1 substantially suppresses Auger recombination, leading to comparable biexciton Auger lifetimes between the two CQD samples. The above observations are in good agreement with the measured PL lifetimes from time-resolved photoluminescence spectroscopy. Increasing the shell CdS thickness while reducing the size of the initial PbS core results in generally longer PL lifetimes (Supporting Information S7).

In addition, comparable with the CdSe/CdS QDs system, we have observed the suppression of the gain threshold upon the epitaxial growth of CdS shell.^48^ Despite current reports of reduced gain thresholds via permanent doping with epitaxial alumina, we have observed reduced gain thresholds in PbS/CdS CQDs with shell-dependent gain characteristics. Therefore, we studied the optical gain of a series of CQDs with the same core of ∼5.9 nm (exciton peak at ∼1550 nm – optimal telecom wavelength) depending on the shell thickness. In Figure 3e and f we show the gain spectra of the three samples with shell thickness of ∼0.2 nm, ∼0.5 nm, and ∼0.8 nm, respectively. We notice a direct dependence of the shell thickness on the gain threshold values, in line with the Auger suppression rates. Thin shells show a gain threshold close to the theoretical values reported for the PbS system, close to ⟨*N*⟩_gain_ = 4.4, while upon the epitaxial growth of thicker shell the gain threshold is reduced to ⟨*N*⟩_gain_ ≈ 3.6 for ∼0.5 nm shell, and down to ⟨*N*⟩_gain_ ≈ 1.7 for ∼0.8 nm shell thickness. It is worth noticing that such suppression of both Auger rates and gain thresholds is stable at room temperature without involving any external doping strategies and encapsulation. To further validate that the PbS/CdS CQDs do not show any doping via ligand exchange we obtain the absorption spectra before and after the solid-state ligand exchange, showing that band-edge absorption does not quench, indicating no evidence of electron doping in the film (Supporting Information S8).

To determine the impact of the shell and its thickness on CQD stability, we measured small clusters of CQDs, depositing the CQD on a glass substrate at ultrahigh dilution levels. We employed an excitation laser power of 1 W/mm^2^ (Supporting Information S9,10). Notably, CQDs of the core/shell series 1—PbS core 1, Sample 1.2 (intermediate shell), and Sample 1.3 (thick shell)—did not undergo any photobleaching for long periods time. On the contrary, all CQDs appeared to undergo modest photobrightening (Supporting Information S9). At this moderate excitation, which was below the saturation regime, the contribution from multiexcitons is not significant.^36^ Therefore, the effect of Auger-mediated nonradiative recombination is not strong. Interestingly, the core-only CQD also exhibits photostability, which can also be attributed by the subsaturation excitation regime under which the measurements were performed, as there is a strong power dependence for photobleaching.^36^ Interestingly, the relative PL enhancement is more pronounced in the case of the PbS core compared to the core/shell CQDs, due to the photoassisted passivation of the trap states under continuous illumination. While various mechanisms have been proposed for CQD photobrightening in air, another mechanism for the photobrightening, as previously proposed,^49^ may also be due to the rearrangement of surface ligands. Fourier transform–infrared (FT-IR) spectroscopy reveals photoinduced changes in the surface chemistry of both PbS and PbS/CdS CQDs films without ligand exchange (Supporting Information S11, Table S11). Upon UV illumination for 30 min, both systems exhibit an increase in the intensities of the asymmetric and symmetric carboxylate stretching vibrations, *v*_asy_(COO^–^) and *v*_sym_(COO^–^), accompanied by a reduction of the broad O–H stretching band associated with protonated carboxylic acid species.^50–54^ This behavior indicates a photoinduced shift from free or weakly associated oleic acid toward deprotonated, surface-coordinated oleate ligands R-COOH → COO^–^···Pb^2+^, which implies improved passivation of undercoordinated Pb surface sites, resulting in a reduced density of trap states and consequently PL enhancement (Supporting Information S9). Notably, these changes are more pronounced in PbS CQDs film, where a larger increase in COO^–^ features and a stronger decrease of the O–H band are observed (Supporting Information S9).

Our work underscores the potential of core/shell heterostructures as an effective strategy to suppress Auger processes when accompanied by interfacial alloying. We demonstrate the synthesis of PbS/CdS core/shell CQDs specifically engineered for telecom-band emission (1500–1620 nm). The CdS shell was grown epitaxially via a cation exchange mechanism, leading to a high crystallinity and monodisperse CQDs. The resulting nanostructures exhibit enhanced optical stability, narrow size distribution, and significantly extended Auger lifetimes while showing a 2-fold reduction of the gain threshold, supporting the feasibility of employing these materials in next-generation NIR light sources. These improvements suggest that engineered core/shell architecture can be used for the realization of lasing in the telecom window by using PbS-based CQDs and bringing CQD-based semiconductor materials closer to real-world optoelectronic applications.

## Supplementary Material



## Notes

Experimental section, characterization methods, additional powder X-ray diffraction (pXRD) spectra and analysis of pXRD patterns of PbS and PbS/CdS CQDs, size distributions, cation exchange reaction kinetics, photoluminescence (PL) and time-resolved photoluminescence (TRPL) spectra (DOCX)

## References

[1] Fan F., Voznyy O., Sabatini R. P., Bicanic K. T., Adachi M. M., McBride J. R., Reid K. R., Park Y.-S., Li X., Jain A., Quintero-Bermudez R., Saravanapavanantham M., Liu M., Korkusinski M., Hawrylak P., Klimov V. I., Rosenthal S. J., Hoogland S., Sargent E. H. (2017). Continuous-Wave Lasing in Colloidal
Quantum Dot Solids Enabled by Facet-Selective Epitaxy. Nature.

[2] Lim J., Park Y.-S., Klimov V. I. (2018). Optical Gain in Colloidal Quantum
Dots Achieved with Direct-Current Electrical Pumping. Nat. Mater..

[3] Geiregat P., Houtepen A. J., Sagar L. K., Infante I., Zapata F., Grigel V., Allan G., Delerue C., Van Thourhout D., Hens Z. (2018). Continuous-Wave Infrared Optical Gain and Amplified Spontaneous Emission
at Ultralow Threshold by Colloidal HgTe Quantum Dots. Nat. Mater..

[4] Klimov V. I., Mikhailovsky A. A., Xu S., Malko A., Hollingsworth J. A., Leatherdale C. A., Eisler H.-J., Bawendi M. G. (2000). Optical Gain and
Stimulated Emission in Nanocrystal Quantum Dots. Science.

[5] Klimov V. I., Ivanov S. A., Nanda J., Achermann M., Bezel I., McGuire J. A., Piryatinski A. (2007). Single-Exciton
Optical Gain in Semiconductor Nanocrystals. Nature.

[6] Jung H., Ahn N., Klimov V. I. (2021). Prospects and Challenges of Colloidal Quantum Dot Laser
Diodes. Nat. Photonics.

[7] Wu K., Park Y.-S., Lim J., Klimov V. I. (2017). Towards Zero-Threshold
Optical Gain Using Charged Semiconductor Quantum Dots. Nature Nanotechnol..

[8] Guzelturk B., Pelton M., Olutas M., Demir H. V. (2019). Giant Modal Gain
Coefficients in Colloidal II-VI Nanoplatelets. Nano Lett..

[9] Wang Y., Zhi M., Chang Y.-Q., Zhang J.-P., Chan Y. (2018). Stable, Ultralow Threshold
Amplified Spontaneous Emission from CsPbBr_3_ Nanoparticles
Exhibiting Trion Gain. Nano Lett..

[10] Schaller R. D., Petruska M. A., Klimov V. I. (2003). Tunable Near-Infrared Optical Gain
and Amplified Spontaneous Emission Using PbSe Nanocrystals. J. Phys. Chem. B.

[11] Hoogland S., Sukhovatkin V., Howard I., Cauchi S., Levina L., Sargent E. H. (2006). A Solution-Processed
1.53 Μm Quantum Dot Laser
with Temperature-Invariant Emission Wavelength. Opt. Express.

[12] Reilly N., Wehrung M., O’Dell R. A., Sun L. (2014). Ultrasmall Colloidal
PbS Quantum Dots. Mater. Chem. Phys..

[13] Dong C., Liu S., Barange N., Lee J., Pardue T., Yi X., Yin S., So F. (2019). Long-Wavelength Lead Sulfide Quantum Dots Sensing up
to 2600 Nm for Short-Wavelength Infrared Photodetectors. ACS Appl. Mater. Interfaces.

[14] Pradhan S., Di Stasio F., Bi Y., Gupta S., Christodoulou S., Stavrinadis A., Konstantatos G. (2019). High-Efficiency Colloidal Quantum
Dot Infrared Light-Emitting Diodes via Engineering at the Supra-Nanocrystalline
Level. Nature Nanotechnol..

[15] Bi Y., Pradhan S., Gupta S., Akgul M. Z., Stavrinadis A., Konstantatos G. (2018). Infrared Solution-Processed
Quantum Dot Solar Cells
Reaching External Quantum Efficiency of 80% at 1.35 Μm and *J*
_sc_ in Excess of 34 mA Cm^–2^. Adv. Mater..

[16] Konstantatos G., Howard I., Fischer A., Hoogland S., Clifford J., Klem E., Levina L., Sargent E. H. (2006). Ultrasensitive Solution-Cast
Quantum Dot Photodetectors. Nature.

[17] Goossens S., Navickaite G., Monasterio C., Gupta S., Piqueras J. J., Perez R., Burwell G., Nikitskiy I., Lasanta T., Galan T., Puma E., Centeno A., Pesquera A., Zurutuza A., Konstantatos G., Koppens F. (2017). Broadband Image Sensor Array Based on Graphene-CMOS
Integration. Nat. Photonics.

[18] Klimov V. I., McGuire J. A., Schaller R. D., Rupasov V. I. (2008). Scaling of Multiexciton
Lifetimes in Semiconductor Nanocrystals. Phys.
Rev. B.

[19] Schaller R. D., Klimov V. I. (2004). High Efficiency Carrier Multiplication
in PbSe Nanocrystals:
Implications for Solar Energy Conversion. Phys.
Rev. Lett..

[20] Ellingson R. J., Beard M. C., Johnson J. C., Yu P., Micic O. I., Nozik A. J., Shabaev A., Efros A. L. (2005). Highly
Efficient
Multiple Exciton Generation in Colloidal PbSe and PbS Quantum Dots. Nano Lett..

[21] Schaller R. D., Sykora M., Pietryga J. M., Klimov V. I. (2006). Seven Excitons at
a Cost of One: Redefining the Limits for Conversion Efficiency of
Photons into Charge Carriers. Nano Lett..

[22] Christodoulou S., Ramiro I., Othonos A., Figueroba A., Dalmases M., Özdemir O., Pradhan S., Itskos G., Konstantatos G. (2020). Single-Exciton
Gain and Stimulated Emission Across
the Infrared Telecom Band from Robust Heavily Doped PbS Colloidal
Quantum Dots. Nano Lett..

[23] Stavrinadis A., Konstantatos G. (2016). Strategies
for the Controlled Electronic Doping of
Colloidal Quantum Dot Solids. ChemPhysChem.

[24] Kroupa D. M., Hughes B. K., Miller E. M., Moore D. T., Anderson N. C., Chernomordik B. D., Nozik A. J., Beard M. C. (2017). Synthesis and Spectroscopy
of Silver-Doped PbSe Quantum Dots. J. Am. Chem.
Soc..

[25] Lu H., Carroll G. M., Chen X., Amarasinghe D. K., Neale N. R., Miller E. M., Sercel P. C., Rabuffetti F. A., Efros A. L., Beard M. C. (2018). *N*-Type PbSe Quantum
Dots via Post-Synthetic Indium Doping. J. Am.
Chem. Soc..

[26] Klimov V. I., Mikhailovsky A. A., McBranch D. W., Leatherdale C. A., Bawendi M. G. (2000). Quantization of Multiparticle Auger Rates in Semiconductor
Quantum Dots. Science.

[27] Kozlov O. V., Park Y.-S., Roh J., Fedin I., Nakotte T., Klimov V. I. (2019). Sub-Single-Exciton
Lasing Using Charged Quantum Dots
Coupled to a Distributed Feedback Cavity. Science.

[28] Cao Y., Stavrinadis A., Lasanta T., So D., Konstantatos G. (2016). The Role of
Surface Passivation for Efficient and Photostable PbS Quantum Dot
Solar Cells. Nature Energy.

[29] Quintero-Bermudez R., Sabatini R. P., Lejay M., Voznyy O., Sargent E. H. (2017). Small-Band-Offset
Perovskite Shells Increase Auger Lifetime in Quantum Dot Solids. ACS Nano.

[30] Taghipour N., Dalmases M., Whitworth G. L., Dosil M., Othonos A., Christodoulou S., Liga S. M., Konstantatos G. (2023). Colloidal
Quantum Dot Infrared Lasers Featuring Sub-Single-Exciton Threshold
and Very High Gain. Adv. Mater..

[31] Cragg G. E., Efros A. L. (2010). Suppression of Auger
Processes in Confined Structures. Nano Lett..

[32] Neo M. S., Venkatram N., Li G. S., Chin W. S., Ji W. (2010). Synthesis
of PbS/CdS Core-Shell QDs and Their Nonlinear Optical Properties. J. Phys. Chem. C.

[33] Neo D. C. J., Cheng C., Stranks S. D., Fairclough S. M., Kim J. S., Kirkland A. I., Smith J. M., Snaith H. J., Assender H. E., Watt A. A. R. (2014). Influence of
Shell Thickness and
Surface Passivation on PbS/CdS Core/Shell Colloidal Quantum Dot Solar
Cells. Chem. Mater..

[34] Ithurria S., Talapin D. V. (2012). Colloidal Atomic
Layer Deposition (c-ALD) Using Self-Limiting
Reactions at Nanocrystal Surface Coupled to Phase Transfer between
Polar and Nonpolar Media. J. Am. Chem. Soc..

[35] Sagar L. K., Walravens W., Zhao Q., Vantomme A., Geiregat P., Hens Z. (2016). PbS/CdS Core/Shell
Quantum Dots by Additive, Layer-by-Layer Shell
Growth. Chem. Mater..

[36] Krishnamurthy S., Singh A., Hu Z., Blake A. V., Kim Y., Singh A., Dolgopolova E. A., Williams D. J., Piryatinski A., Malko A. V., Htoon H., Sykora M., Hollingsworth J. A. (2021). PbS/CdS
Quantum Dot Room-Temperature Single-Emitter Spectroscopy Reaches the
Telecom O and S Bands via an Engineered Stability. ACS Nano.

[37] Tahara H., Sakamoto M., Teranishi T., Kanemitsu Y. (2018). Quantum Coherence
of Multiple Excitons Governs Absorption Cross-Sections of PbS/CdS
Core/Shell Nanocrystals. Nat. Commun..

[38] Tahara H., Sakamoto M., Teranishi T., Kanemitsu Y. (2017). Harmonic Quantum
Coherence of Multiple Excitons in PbS/CdS Core-Shell Nanocrystals. Phys. Rev. Lett..

[39] Hines M. A., Scholes G. D. (2003). Colloidal PbS Nanocrystals
with Size-Tunable Near-Infrared
Emission: Observation of Post-Synthesis Self-Narrowing of the Particle
Size Distribution. Adv. Mater..

[40] Moreels I., Lambert K., Smeets D., De Muynck D., Nollet T., Martins J. C., Vanhaecke F., Vantomme A., Delerue C., Allan G., Hens Z. (2009). Size-Dependent
Optical Properties of Colloidal PbS Quantum Dots. ACS Nano.

[41] Pietryga J. M., Werder D. J., Williams D. J., Casson J. L., Schaller R. D., Klimov V. I., Hollingsworth J. A. (2008). Utilizing the Lability of Lead Selenide
to Produce Heterostructured Nanocrystals with Bright, Stable Infrared
Emission. J. Am. Chem. Soc..

[42] Zhao H., Chaker M., Wu N., Ma D. (2011). Towards Controlled
Synthesis and Better Understanding of Highly Luminescent PbS/CdS Core/Shell
Quantum Dots. J. Mater. Chem..

[43] Lambert K., Geyter B. D., Moreels I., Hens Z. (2009). PbTe|CdTe Core|Shell
Particles by Cation Exchange, a HR-TEM Study. Chem. Mater..

[44] Casavola M., Van Huis M. A., Bals S., Lambert K., Hens Z., Vanmaekelbergh D. (2012). Anisotropic Cation Exchange in PbSe/CdSe Core/Shell
Nanocrystals of Different Geometry. Chem. Mater..

[45] Torquato S., Jiao Y. (2009). Dense Packings of Polyhedra:
Platonic and Archimedean Solids. Phys. Rev.
E.

[46] Dzhagan V. M., Valakh M. Ya., Milekhin A. G., Yeryukov N. A., Zahn D. R. T., Cassette E., Pons T., Dubertret B. (2013). Raman- and
IR-Active Phonons in CdSe/CdS Core/Shell Nanocrystals in the Presence
of Interface Alloying and Strain. J. Phys. Chem.
C.

[47] Tschirner N., Lange H., Schliwa A., Biermann A., Thomsen C., Lambert K., Gomes R., Hens Z. (2012). Interfacial Alloying
in CdSe/CdS Heteronanocrystals: A Raman Spectroscopy Analysis. Chem. Mater..

[48] Bisschop S., Geiregat P., Aubert T., Hens Z. (2018). The Impact of Core/Shell
Sizes on the Optical Gain Characteristics of CdSe/CdS Quantum Dots. ACS Nano.

[49] Jones M., Nedeljkovic J., Ellingson R. J., Nozik A. J., Rumbles G. (2003). Photoenhancement
of Luminescence in Colloidal CdSe Quantum Dot Solutions. J. Phys. Chem. B.

[50] Deacon G. B., Phillips R. J. (1980). Relationships between
the Carbon-Oxygen Stretching
Frequencies of Carboxylato Complexes and the Type of Carboxylate Coordination. Coord. Chem. Rev..

[51] Cass L. C., Malicki M., Weiss E. A. (2013). The Chemical
Environments of Oleate
Species within Samples of Oleate-Coated PbS Quantum Dots. Anal. Chem..

[52] Kennehan E. R., Munson K. T., Doucette G. S., Marshall A. R., Beard M. C., Asbury J. B. (2020). Dynamic Ligand Surface
Chemistry of Excited PbS Quantum
Dots. J. Phys. Chem. Lett..

[53] Sowa J. K., Roberts S. T., Rossky P. J. (2023). Exploring
Configurations of Nanocrystal
Ligands Using Machine-Learned Force Fields. J. Phys. Chem. Lett..

[54] Zhang J., Zhang H., Cao W., Pang Z., Li J., Shu Y., Zhu C., Kong X., Wang L., Peng X. (2019). Identification
of Facet-Dependent Coordination Structures of Carboxylate Ligands
on CdSe Nanocrystals. J. Am. Chem. Soc..

