# Supplementary material for: Synthesis of Monodisperse PbS/CdS Colloidal Quantum Dots Emitting at Telecommunication Wavelengths with suppressed Auger Rates and Gain Threshold
Source: Nano Lett. 2026 Feb 26;26(9):3266–73. doi: 10.1021/acs.nanolett.6c00158 (PMC12983354; doi:10.1021/acs.nanolett.6c00158)
Supplement: Supplementary file 1 [file nl6c00158_si_001.docx]

**Supporting Information for:**

**Synthesis of Monodisperse PbS/CdS Colloidal Quantum Dots Emitting at Telecommunication Wavelengths with suppressed Auger Rates and Gain Threshold**

Marios Stylianou^1^, Eric G. Bowes^2^, Luca Leoncino^3^, Rosaria Brescia^3^, Victoria Nisoli^2^, Nathan A. Malone^2^, Jessica Q. Geisenhoff^2^, Jennifer A. Hollingsworth^2^, Andreas Othonos^4^, Sotirios Christodoulou*^,1^

^1^Inorganic Nanocrystals Laboratory, Department of Chemistry, University of Cyprus, Nicosia 1678, Cyprus

^2^Materials Physics & Applications Division: Center for Integrated Nanotechnologies, Los Alamos National Laboratory, Los Alamos, New Mexico 87545, USA

^3^Electron Microscopy Facility, Instituto Italiano di Tecnologia, Genova 16163, Italy

^4^Laboratory of Ultrafast Science, Department of Physics, University of Cyprus, Nicosia 2109, Cyprus

**Experimental section**

**Chemicals**

Lead (II) oxide (PbO, 99.999%), oleic acid (OA, technical grade 90%), 1-octadecene (ODE, technical grade 90%), hexamethyldisilathiane ((TMS)_2_S, synthesis grade), 1-ethyl-3-methylimidazolium (EMII, 97%) and 3-mercaptopropionic acid (MPA, ≥99%) were purchased from Sigma-Aldrich. Cadmium (II) oxide (CdO, 99.998%) was purchased from Thermo Scientific Chemicals. Tetrachloroethylene (TCE, 99.9%) was purchased from abcr. Anhydrous methanol (MeOH, ≥99.9%) and anhydrous toluene (C_7_H_8_, ≥99.8%) were purchased from CARLO ERBA Reagents.

**PbS CQDs synthesis**

For the PbS CQDs synthesis, briefly 448 mg lead (II) oxide (PbO), 3.8 mL oleic acid (OA) and 50 mL 1-octadecene (ODE) were stirred in a 100 mL three-necked rounded flask at room temperature. Then the mixture was heated at 120 ^o^C under vacuum for 2 h to form a clear solution of lead (II) oleate [Pb(oleate)_2_]. Setting the temperature at 115 ^o^C, we quickly injected under N_2_ a solution of 60 μL hexamethyldisilathiane [(TMS)_2_S] dissolved in 0.5 mL degassed ODE letting the reaction to take place for about thirty minutes. Keeping the same parameters of the first injection (temperature, concentration of S-precursor, reaction time) we optimized the reaction carrying out multiple injections of (TMS)_2_S / ODE to achieve the desired sizes of PbS CQDs. At the end of the reaction, the solution was cooled down with air, quenched with a mixture of acetone / ethanol and washed three times with a mixture of acetone / ethanol, redispersing in anhydrous toluene.

**PbS/CdS CQDs core/shell series synthesis**

To create the three PbS/CdS core/shell series, 1.5 mL of a stock solution of 0.3 M cadmium (II) oleate [Cd(oleate)_2_] in ODE (θ = 50 ^o^C.) were injected under N_2_ in a total volume of 3 mL of PbS CQDs dispersed in toluene (⁓5.7 μM of PbS CQDs) at 100 ^o^C. Cd-oleate stock solution was prepared by mixing briefly 1.0 g cadmium (II) oxide (CdO), 6.0 mL oleic acid (OA) and 22 mL 1-octadecene (ODE) in a 50 mL three-necked rounded flask. The mixture was heated to 255 ^o^C under N_2_ with vigorous stirring, resulting in a clear solution. Then, the clear solution was cooled down to 50 ^o^C and was left under vacuum for further use. Using three different sized PbS CQDs, we synthesized three PbS/CdS core/shells series via cation exchange tuning the reaction’s time from 2 min to 50 h and introducing the required Cd-precursor to achieve the desired core/shells. For the synthesis of Samples 3.3 and 3.4, we performed two and three injections of 0.3 M Cd(oleate)_2_, respectively. At the end of the reaction, the solution was cooled down with ice bath and washed two times with acetone and ethanol, redispersing in anhydrous toluene.

**Fabrication of PbS and PbS/CdS CQDs films**

The PbS and PbS/CdS CQDs (30 mg/mL) were spin-cast onto glass substrate at the speed rate of 2500 rpm for 20s. Subsequently, a solution of EMII (7 mg/mL ZnI_2_ in anhydrous methanol) containing 0.03% MPA was applied to the solid film surface to facilitate ligand exchange and enhance CQD passivation by reducing surface defects. After 30 s, the spin-coater was started again to dry the film, while a few drops of anhydrous methanol were drop-casted to remove remain organics. This procedure repeated until a thick film was achieved (6 layers). The thickness of the films was measured using a profilometer to calculate <N> values for each sample.

**Transient absorption (TA) measurements**

Transient absorption measurements were carried out using a titanium sapphire based ultrafast amplifier centred at 800 nm and generating 45 fs pulses at a repetition rate of 1 kHz. The optical setup utilized was a typical pump-probe non-collinear configuration. The main part of the fundamental energy from the amplifier was directed into a half wave plate and a thin film polarizer system to control the energy of the excitation pulse incident of the sample (0.5-50 μJ/pulse). The optical path of the pump beam included an optical chopper allowing the use of phase-sensitive detection thereby improving the signal-to-noise ratio. An optical parametric amplifier pumped with approximately 1 mJ of the fundamental 800 nm energy was used to generate the probe beam with wavelengths ranging from 1200 nm to 1700 nm. The probe beam’s optical path included a precise motorized translation stage to control the optical delay between the pump and the probe beam. The probe beam was directed on the sample within the excitation area of the pump beam where changes in transmission and reflection were recorded simultaneously using lock-in amplifiers. From the data analysis, the biexciton Auger lifetimes τ_xx_ were calculated for all samples.

**Absorption measurements.** Room-temperature absorption measurements were taken using a Shimadzu, UV-3600 UV-Vis-NIR spectrometer. Spectra were obtained in TCE for the range of 800-2000 nm.

**FT-IR measurements.** Room-temperature FT-IR measurements were taken using a Shimadzu FT-IR spectrometer equipped with a QATR-S ATR accessory. Spectra were obtained on solid-state PbS and PbS/CdS CQDs films for the range of 400-4000 cm^-1^.

**Widefield Microscopy**. Samples were prepared by drop-casting dilute toluene solutions of PbS and PbS/CdS QDs onto clean coverslips. Although single-dot measurements were not possible under these experimental conditions, TEM studies were performed utilizing identical drop-casting conditions to confirm that the QDs were distributed primarily as single dots and small clusters on the surface. PL studies were performed on a home-built wide-field fluorescence microscope with an excitation of 488 nm continuous wave (cw) laser. PL emission was collected with an IR objective lens (50x/ NA0.65, Olympus), cleaned with long-pass filters, and directed to a Princeton NIRvana:640 charge-coupled-device (CCD) camera. Excitation was achieved using an average power density of 1 W/mm^2^. A time series of image frames was collected under constant illumination with an integration time of 10 s per frame for a total acquisition time of ∼30-60 min. ImageJ was used to define regions of interest (ROIs) within the PL images for quantitative analysis of fluorescence intensity.

**Ensemble QD Optical Characterization**. *Absorption and PL Spectroscopy:* Stock solutions of QDs in toluene were diluted in tetrachloroethylene (TCE) to suitable optical densities for absorption and PL measurements. Absorption spectra were recorded using a Cary 5000 Spectrometer. The PL spectra were recorded using an Edinburgh Instruments FLS1000 spectrometer equipped with an InP/InGaAs photomultiplier tube (PMT). *Quantum Yields*: Quantum Yields were determined using direct methods using 800 nm excitation in an Edinburgh Instruments FLS1000 Spectrometer equipped with an integrating sphere. The samples were diluted to achieve an optical density (OD) of 0.1 at the excitation wavelength, minimizing reabsorption artifacts. *Excited-State Lifetimes*. The samples for the lifetime measurements were prepared in the same way as for the quantum-yield measurements. The measurements were performed using a multi-channel scaling (MCS) module in an Edinburgh Instruments FLS1000 spectrophotometer equipped with a Liquid-nitrogen cooled Near-IR PMT (Hamamatsu R5509-73) and an Edinburgh Instruments VPL-405 pulsed laser diode with peak emission at 405 nm (pulse width of 200 ns). For each sample, the PL decays were recorded at the emission wavelength maxima.

**Structural characterization.** Powder X-ray diffractograms were measured with a Rigaku Miniflex 6G X-ray diffractometer (CuKa, λ = 1.5418Å). TEM samples were prepared by drop-casting onto ultrathin-on-holey carbon film on a Cu grid. High-resolution TEM (HRTEM) and high-angle annular dark field- scanning TEM (HAADF-STEM) imaging were carried out using an image-Cs-corrected JEM-2200FS TEM, operated at 200 kV. In order to slow-down carbon contamination under the e-beam, HRTEM images were acquired in low-dose-rate conditions, using a direct electron detection camera (Gatan K2 Summit). The dose rate used is about 10 times lower than what needed for HRTEM image acquisition using a typical scintillator-coupled CCD camera. The images shown here are ROIs of wide field of view ((140 nm)^2^) frames, each of them obtained by cross-correlation-alignment and summed frames obtained by short exposure time (0.3 s), up to a total exposure time of 12 s. Mean dilation maps were obtained here using the peak pairs analysis (PPA) method^1^ on the HRTEM images of individual core-shell particles, with the aim of identifying the position of the PbS core. The HRTEM images shown here were Fourier-filtered, using an average background subtraction filter (ABSF), to minimize the contribution to the contrast from the amorphous component.^2^

Elemental mapping was done using a Bruker XFlash-5060 SDD-based energy-dispersive X-ray spectrometry (EDS) system. To slow down C-contamination during the prolonged acquisition, the STEM-EDS maps were acquired with the sample cooled to -35 °C using a cryo-transfer sample holder (Gatan, mod. 626). The STEM-EDS line scan shown here was obtained via processing of the original STEM-EDS map: first the map was binned over the spatial coordinate perpendicular to the line scan direction (vertical direction in Fig. 2e). In a second step, the EDS counts were summed to obtain the elemental profiles for Pb and Cd: Pb_Lα and Pb_Lβ1 peaks were taken in account for Pb (in red) while Cd_Lα, Cd_Lβ1 and Cd_Lβ2 peaks were summed for Cd (in green). Data processing was performed by the means of Hyperspy, an open source Pyhton library written for the analysis of multidimensional datasets such as STEM-EDS data.^3^

**S1: Powder X-ray diffraction (XRD) spectra of PbS/CdS core/shell series**

**a**

**
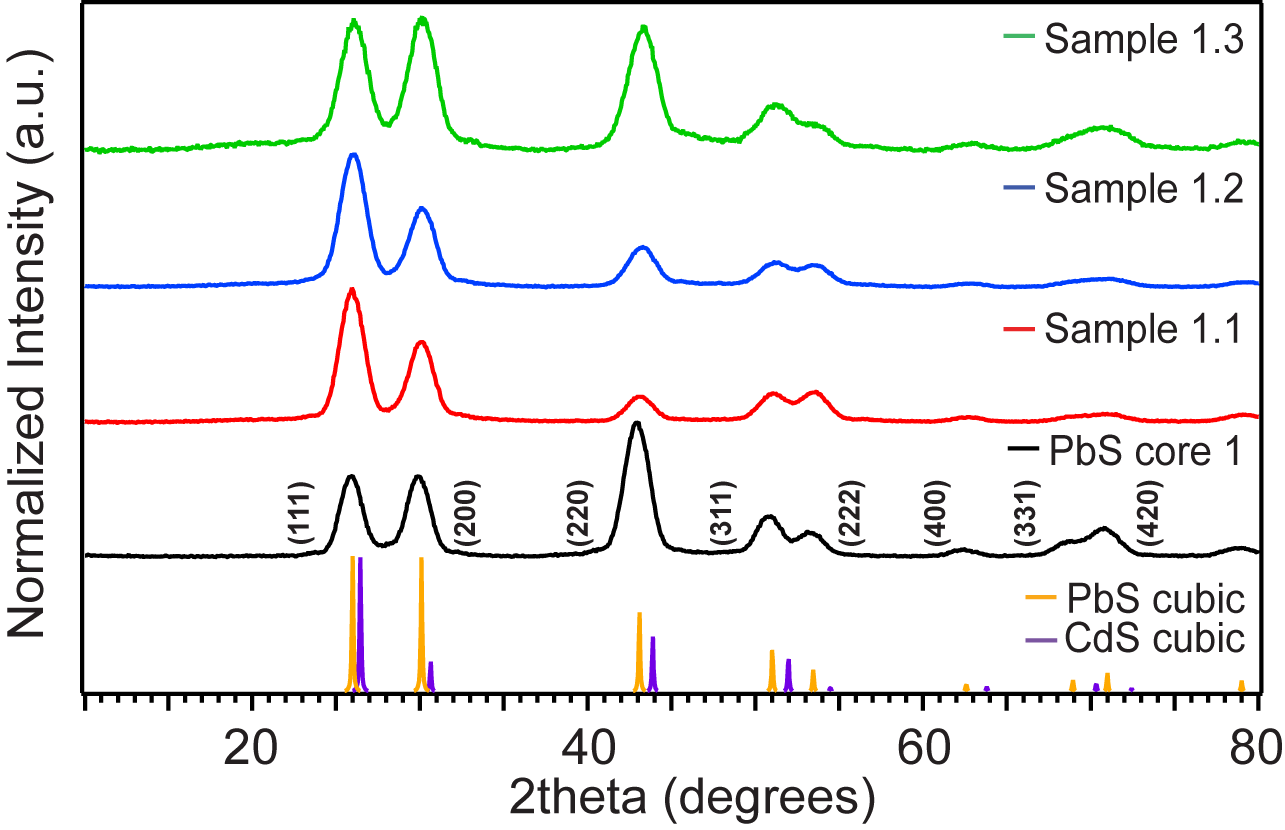
**

**b**

**
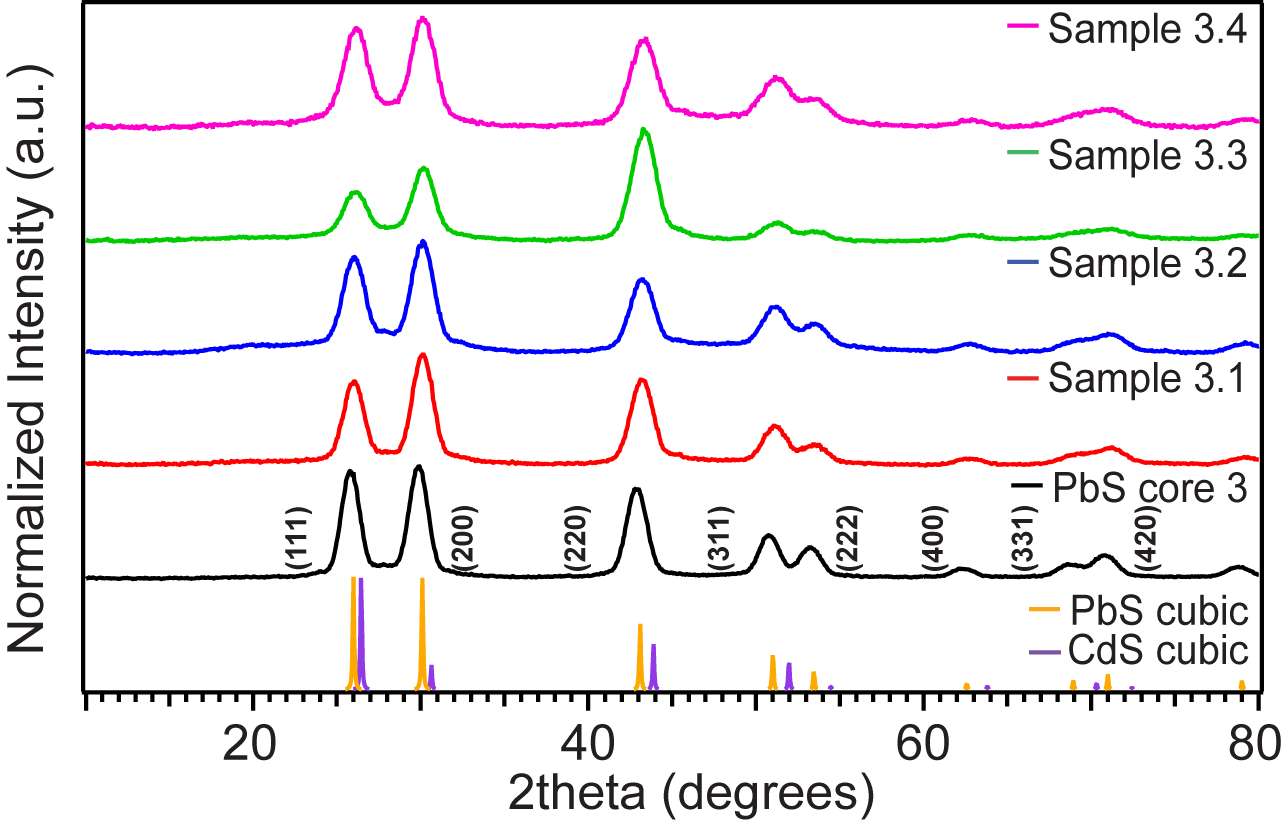
**

**Figure S1-1:** Powder X-ray diffraction spectra (XRD) of PbS/CdS core/shell series: a. Core/shell series 1 and b. Core/shell series 3. Theoretical patterns of cubic rock-salt PbS (COD No. 5000087) and cubic zinc blende CdS (COD No. 9008839).

**Table S1.** Closer analysis of pXRD patterns of PbS core 2 and Sample 2.3. 2theta values of peaks’ position corresponding to the respective crystal planes, Full Width Half Maximum (FWHM) and distribution σd.

| **Crystal planes**  **(hkl)** | **PbS core 2** | | | **Sample 2.3** | |
| --- | --- | --- | --- | --- | --- |
|  | **2theta (degrees)** | | **FWHM / σd (degrees / %)** | **2theta (degrees)** | **FWHM / σd (degrees / %)** |
| (111) | 25.8 | 1.53 / 0.33 | | 26.1 | 1.76 / 0.87 |
| (200) | 29.8 | 1.56 / 0.20 | | 30.2 | 1.74 / 0.93 |
| (220) | 42.8 | 1.63 / 0.28 | | 43.3 | 1.94 / 1.38 |
| (311) | 50.7 | 1.60 / 0.55 | | 51.2 | 1.61 / 4.31 |
| (222) | 53.2 | 1.79 / 0.99 | | 53.4 | 2.49 / 13.0 |
| (400) | 62.5 | 2.02 / 1.57 | | 62.7 | 2.02 / 6.61 |
| (331) | 68.6 | 2.11 / 1.63 | | 69.5 | 2.76 / 34.3 |
| (420) | 70.8 | 1.93 / 0.83 | | 71.6 | 2.33 / 23.7 |

**S2: Transmission Electron Microscopy (TEM) images**

**PbS/CdS core/shell series 1**

**a**

**
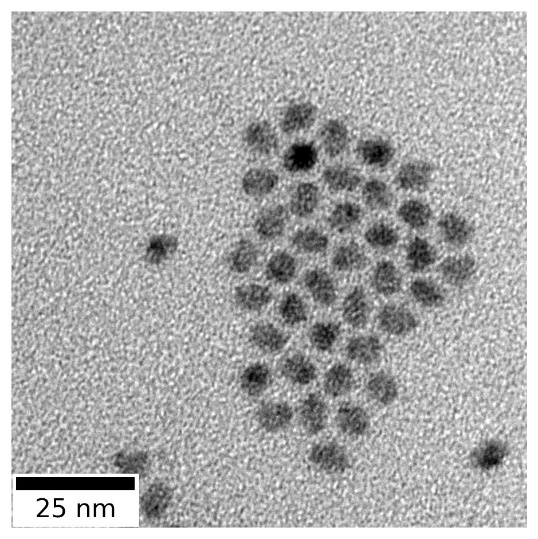

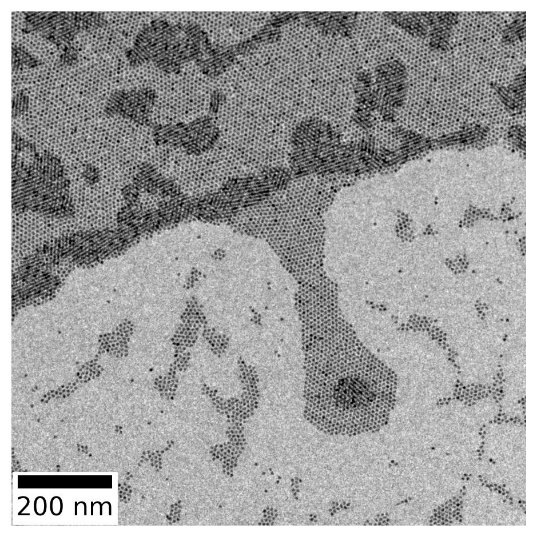
**

**b**

**
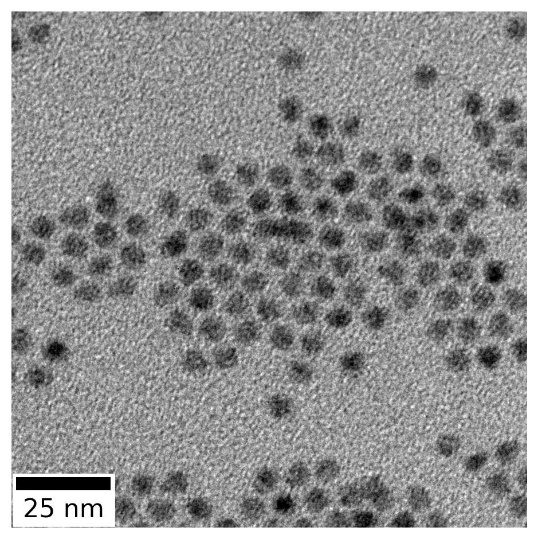
**
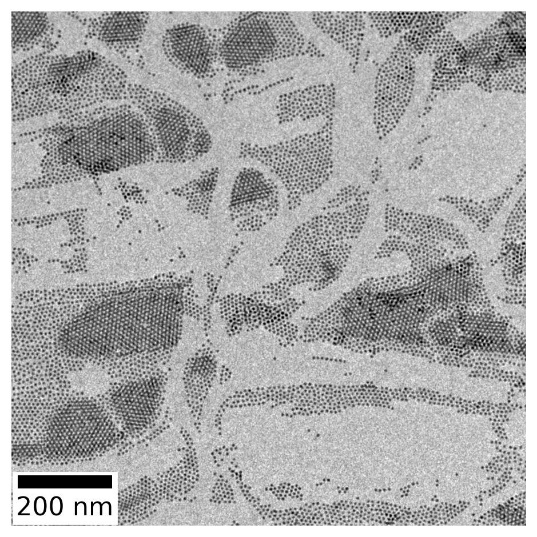


**c**


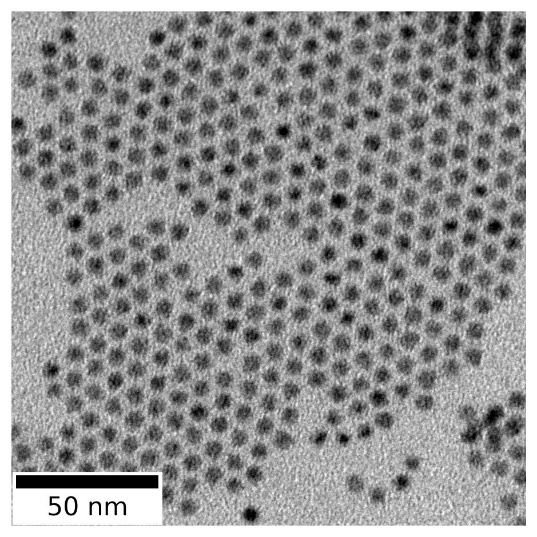
 **
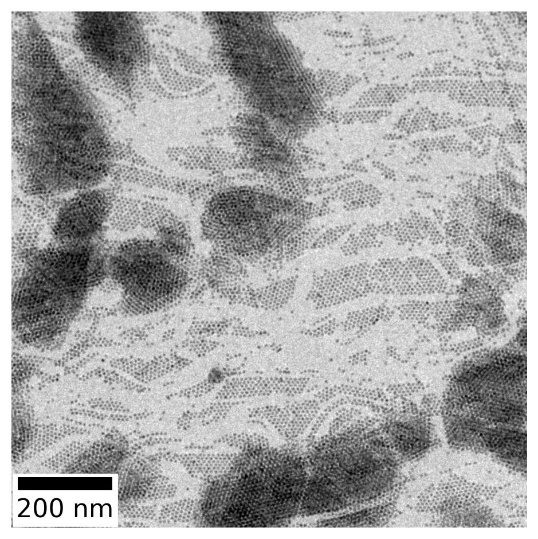
**

**d**

**
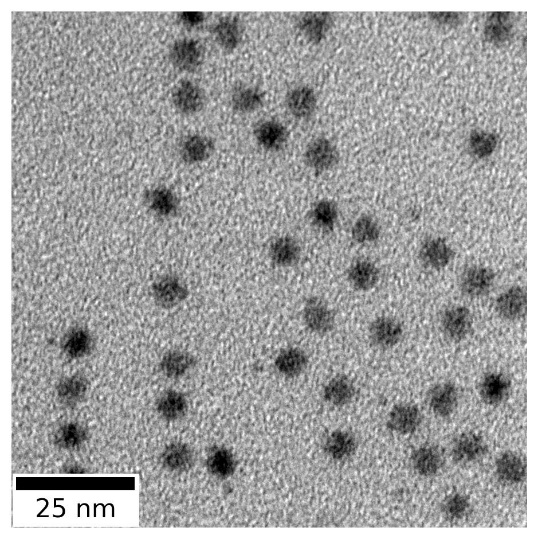
**
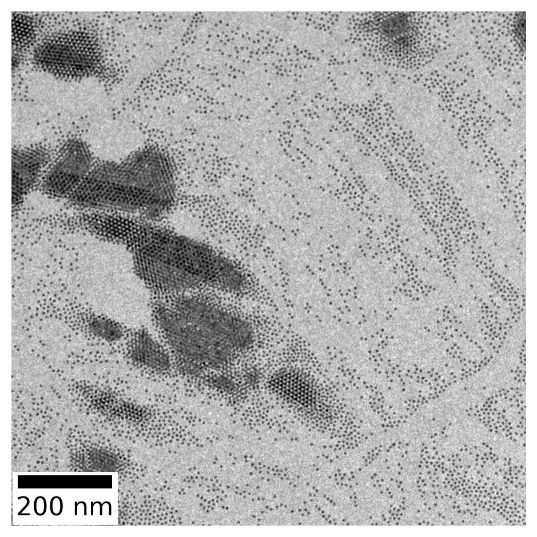


**Figure S2-1:** TEM images of PbS/CdS core/shell series 1: a. PbS core 1, b. Sample 1.1, c. Sample 1.2, and d. Sample 1.3.

**PbS/CdS core/shell series 2**

**a**


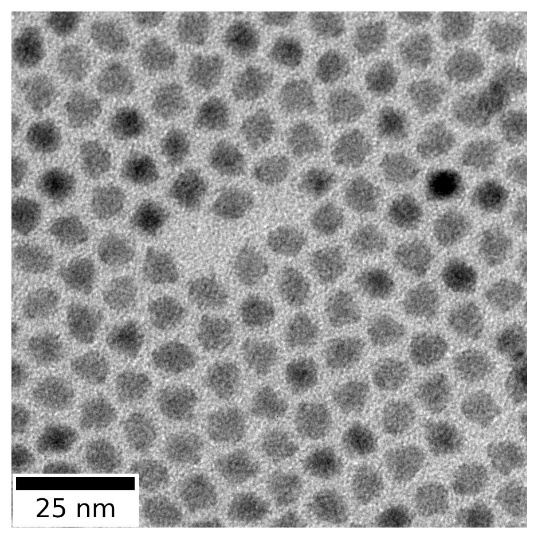

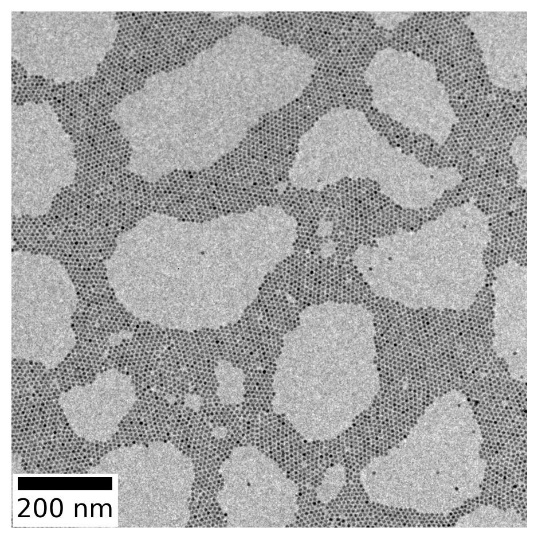


**b**

**
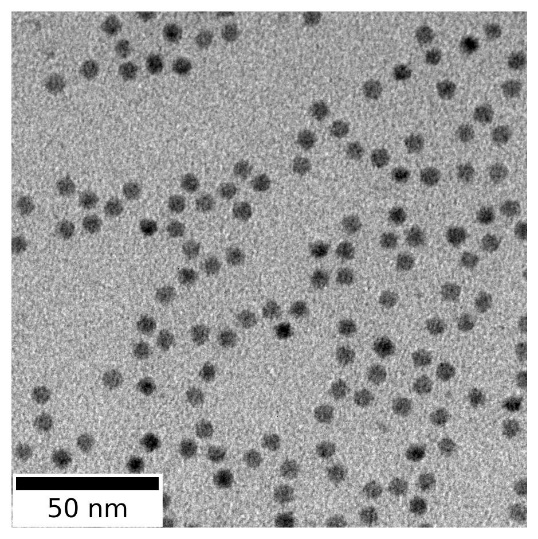
**
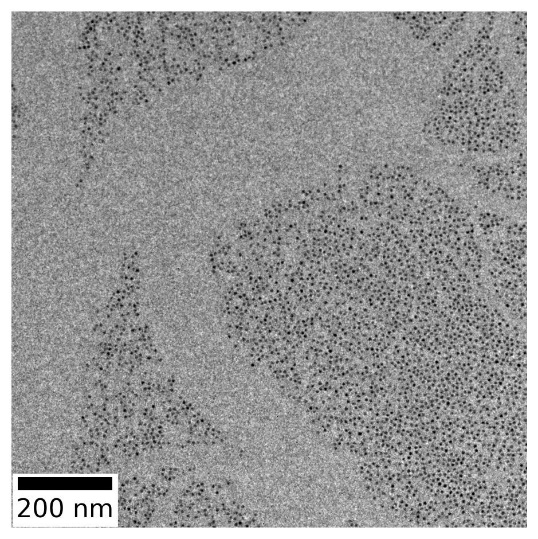


**c**

**
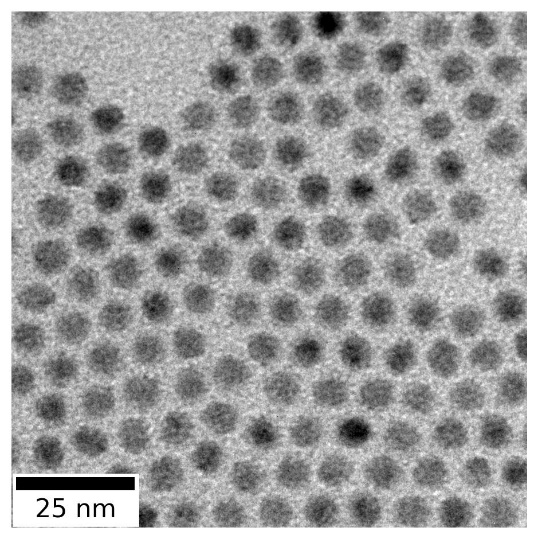
**
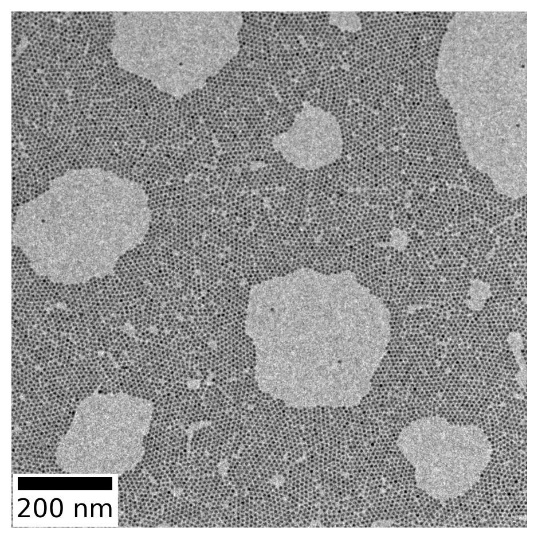


**d**

**
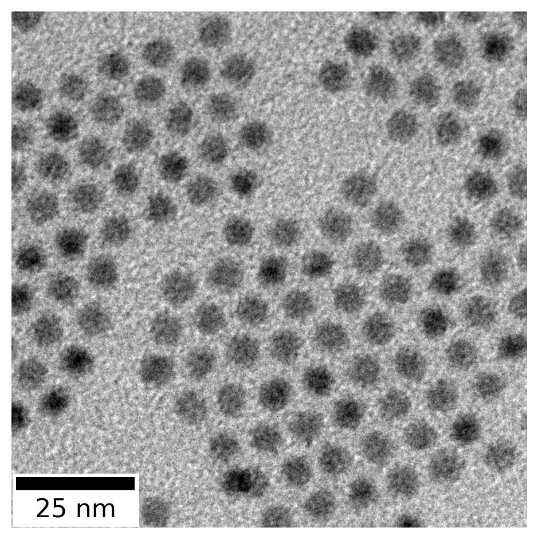

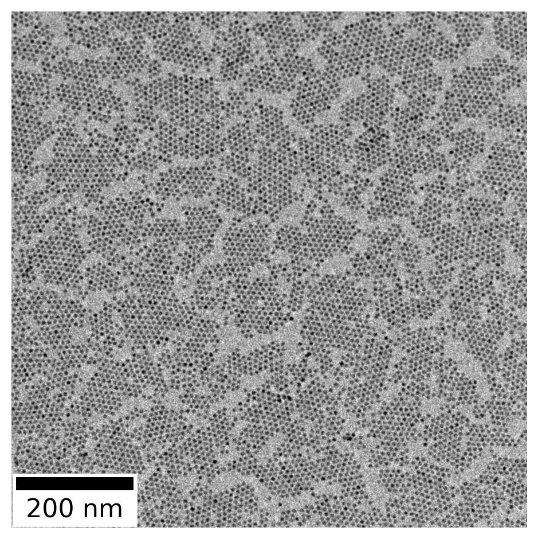
**

**Figure S2-2:** TEM images of PbS/CdS core/shell series 2: a. PbS core 2, b. Sample 2.1, c. Sample 2.2, and d. Sample 2.3.

**PbS/CdS core/shell series 3**

**a**

**
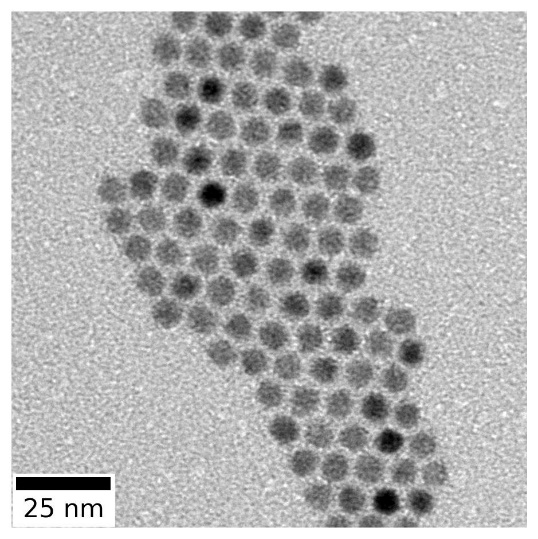

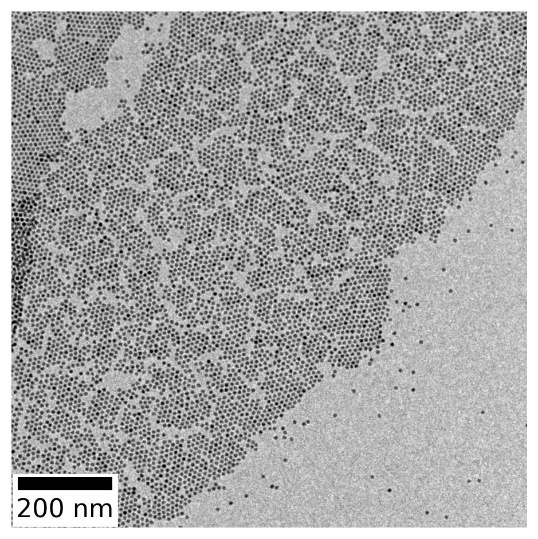
**

**b**

**
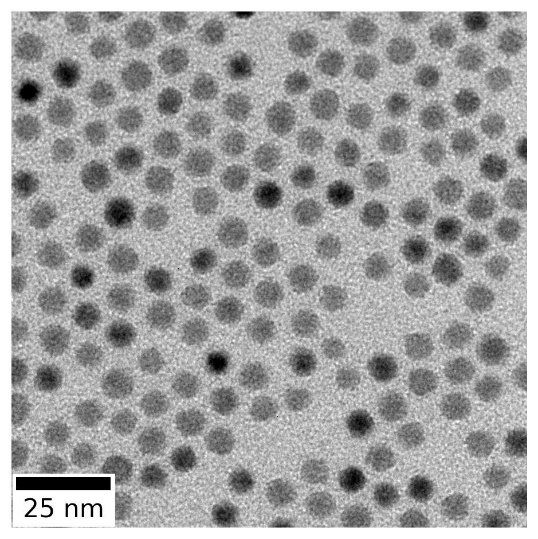
** **
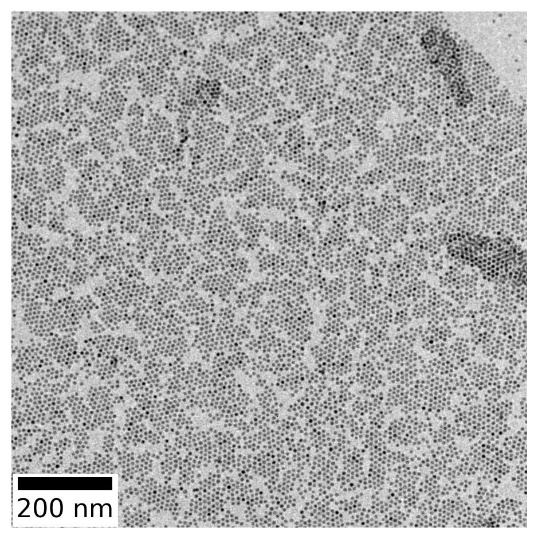
**

**c**

**
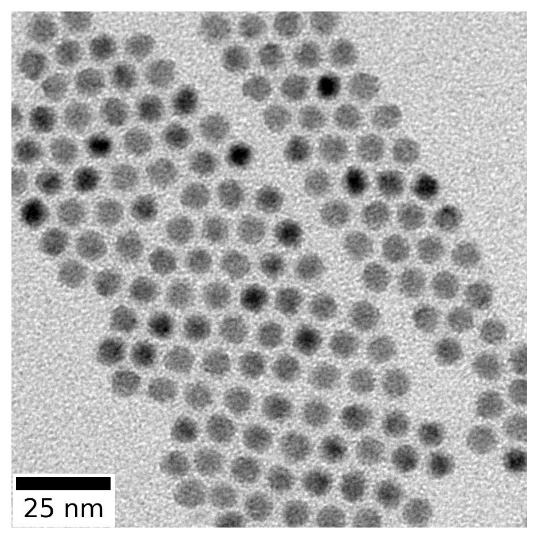
** **
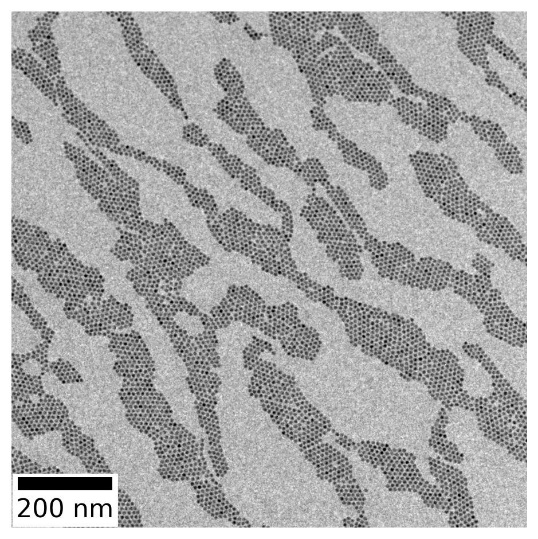
**

**d**

**
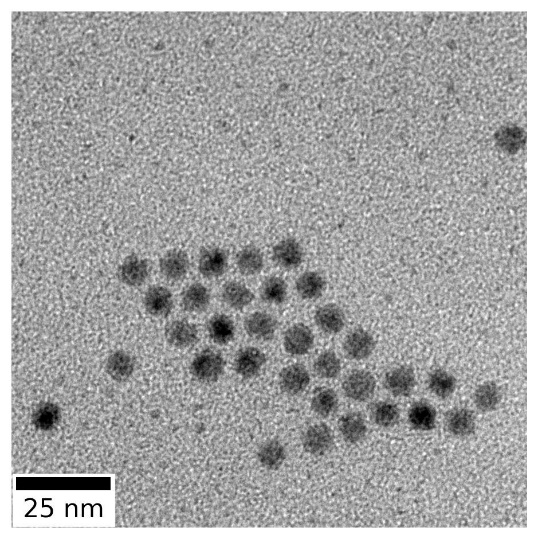

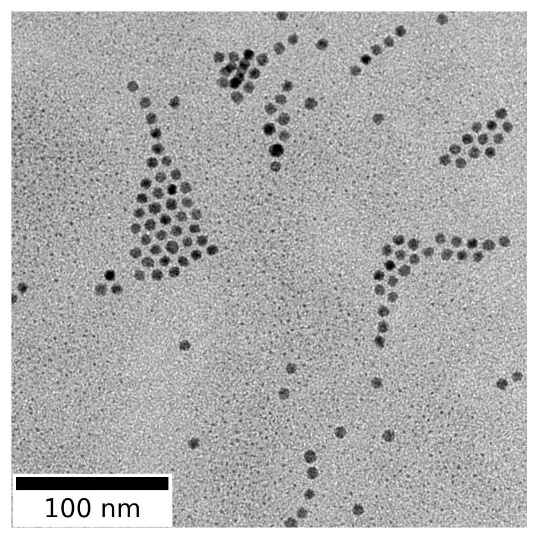
**

**Figure S2-3:** TEM images of PbS/CdS core/shell series 3: a. PbS core 3, b. Sample 3.1, c. Sample 3.2, and d. Sample 3.3.

**S3: Size distribution**

**PbS/CdS core/shell series 1**

**a b**

**
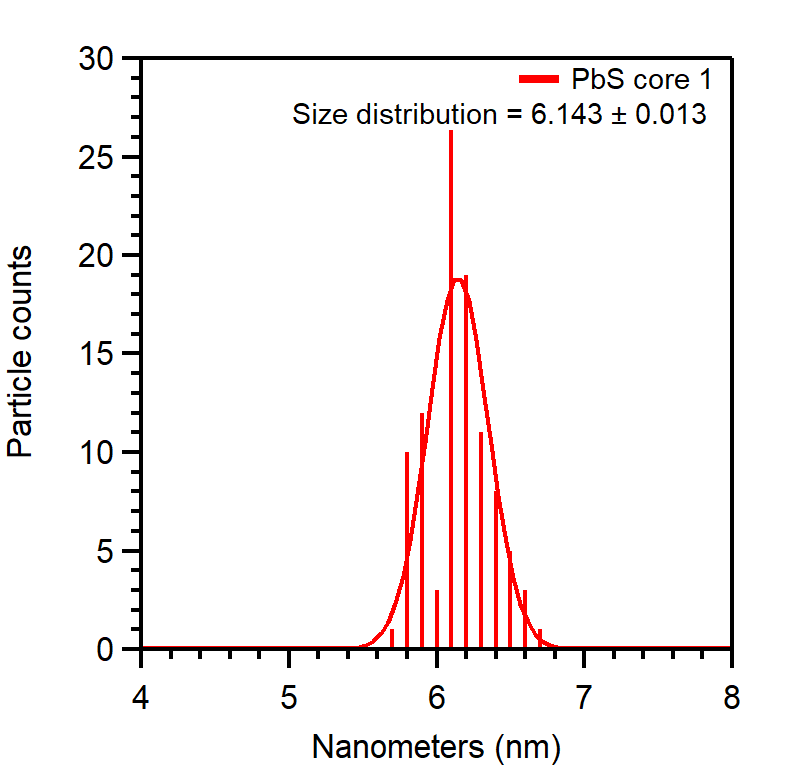

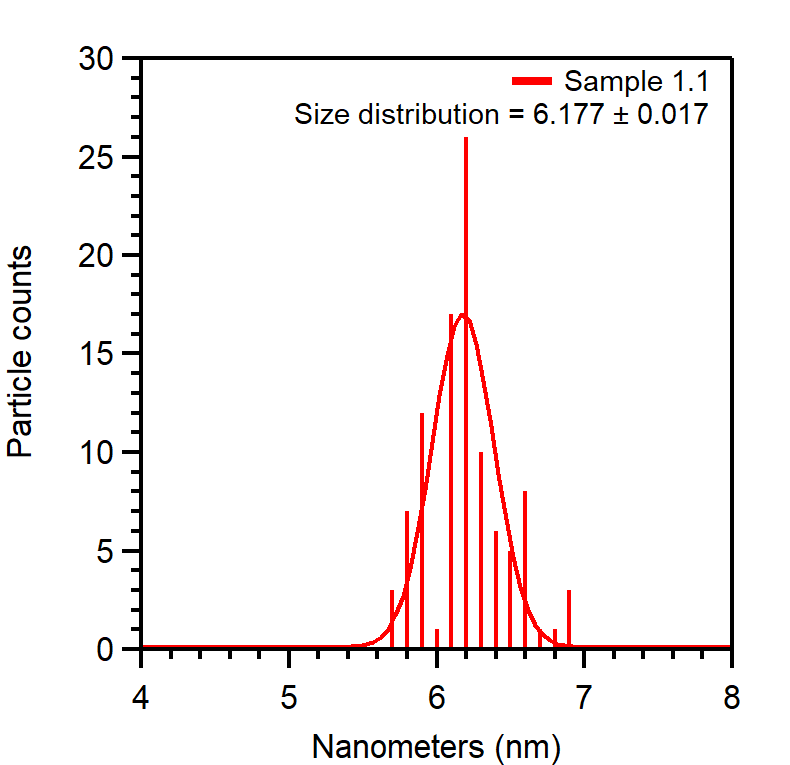
**

**c d**

**
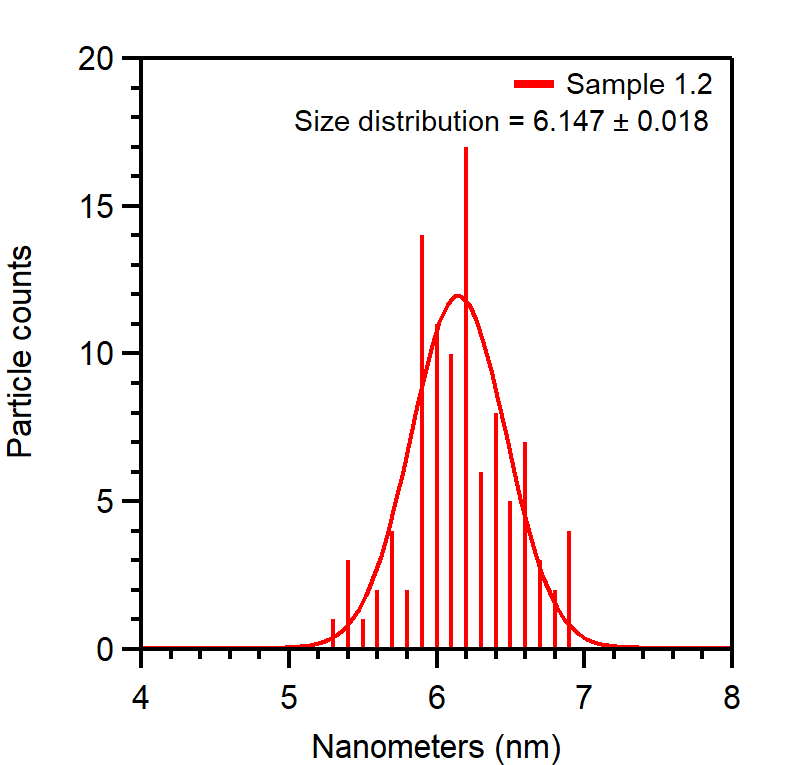

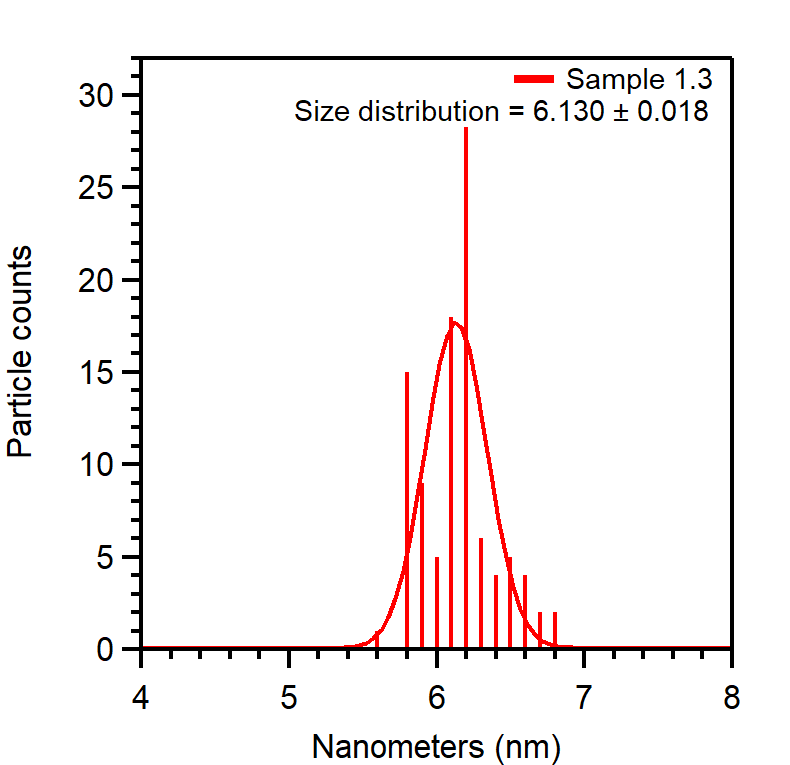
**

**Figure S3-1:** Size distribution of PbS/CdS core/shell series 1: a. PbS core 1, b. Sample 1.1, c. Sample 1.2, and d. Sample 1.3.

**PbS/CdS core/shell series 2**

**a b**

**
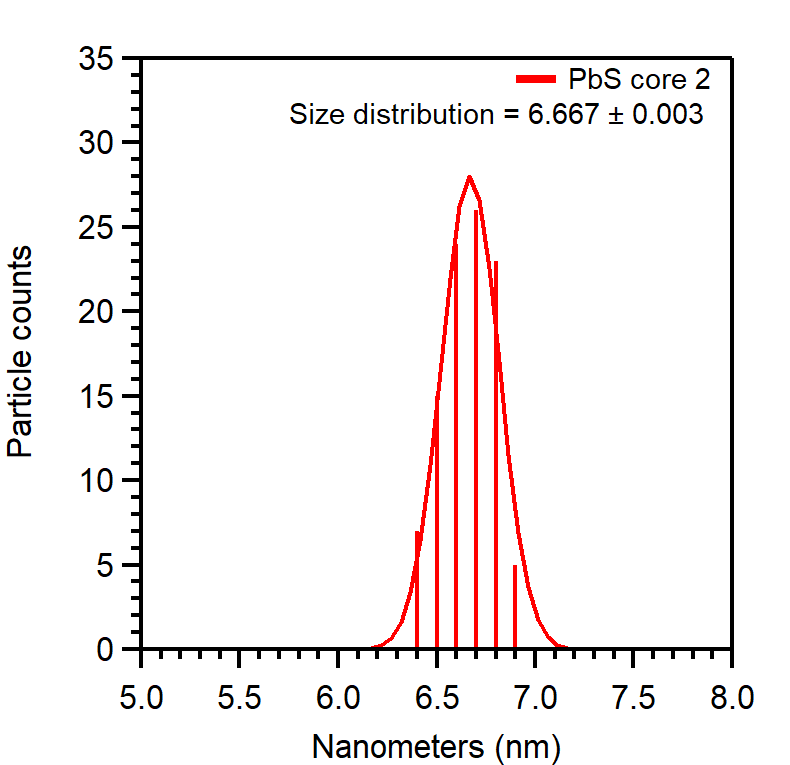

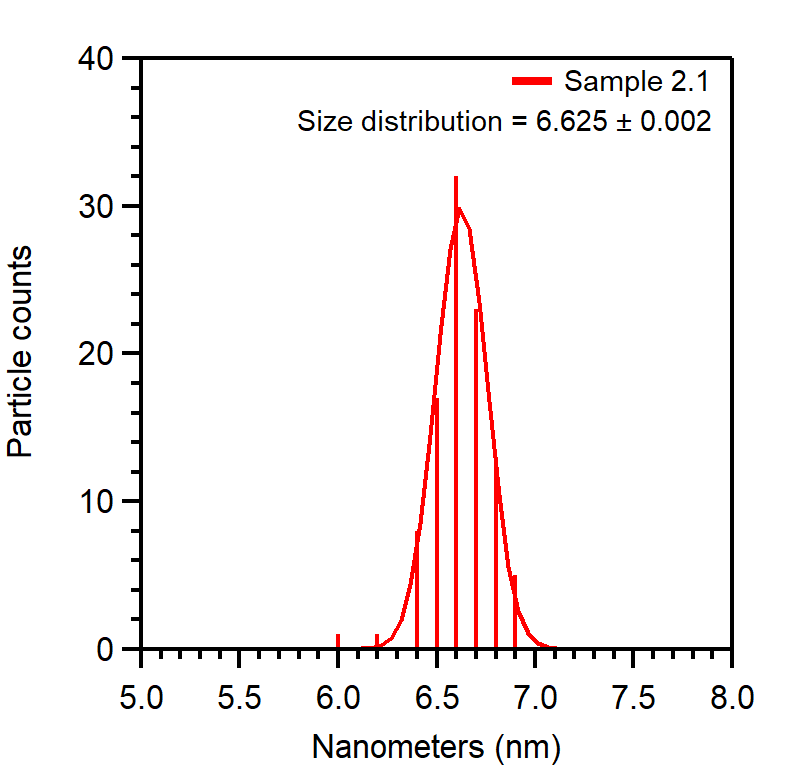
**

**c d**

**
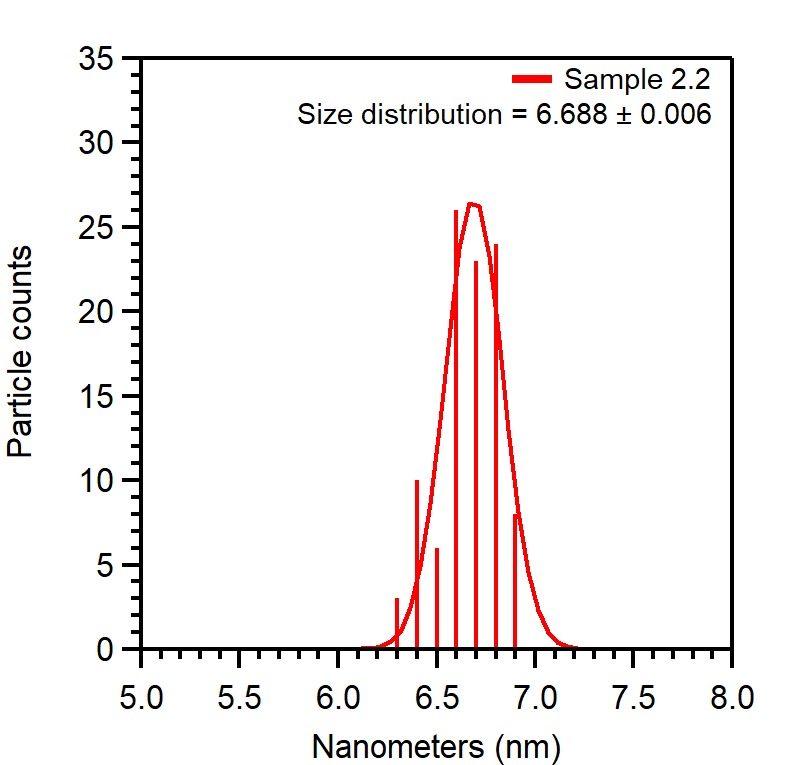

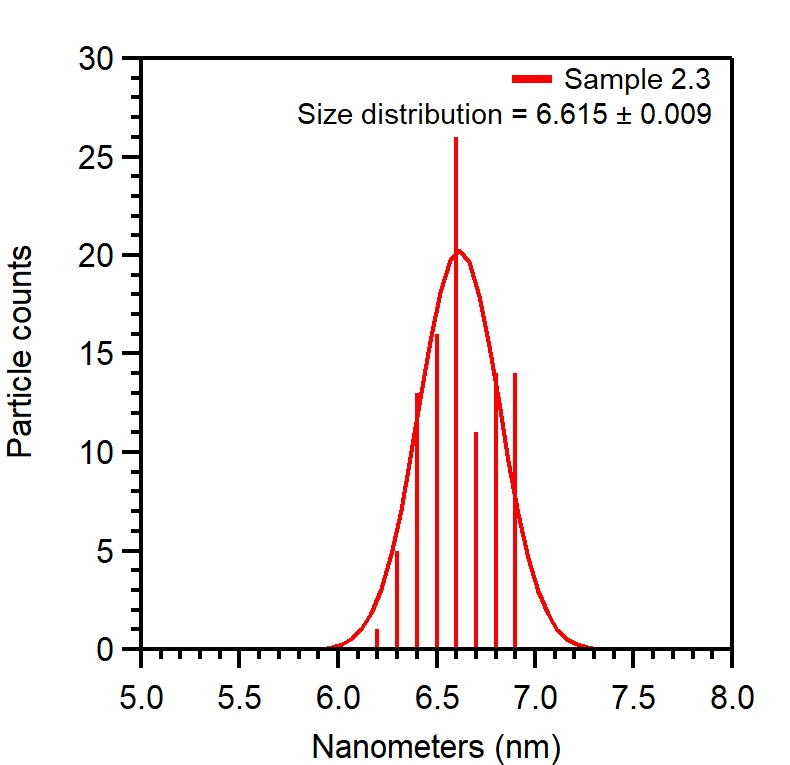
**

**Figure S3-2:** Size distribution of PbS/CdS core/shell series 2: a. PbS core 2, b. Sample 2.1, c. Sample 2.2, and d. Sample 2.3.

**PbS/CdS core/shell series 3**

**a b**

**
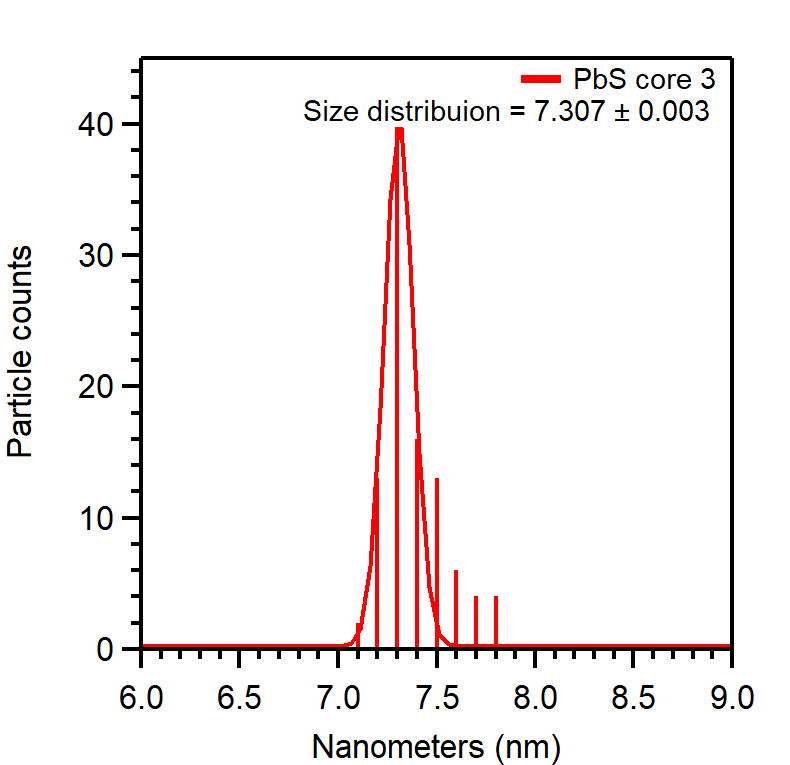

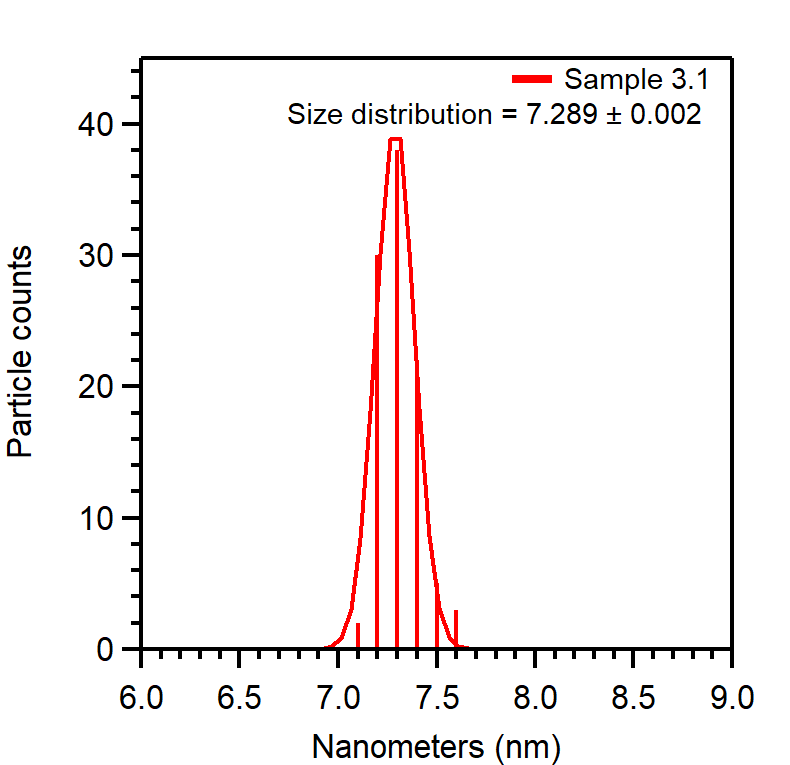
**

**c d**

**
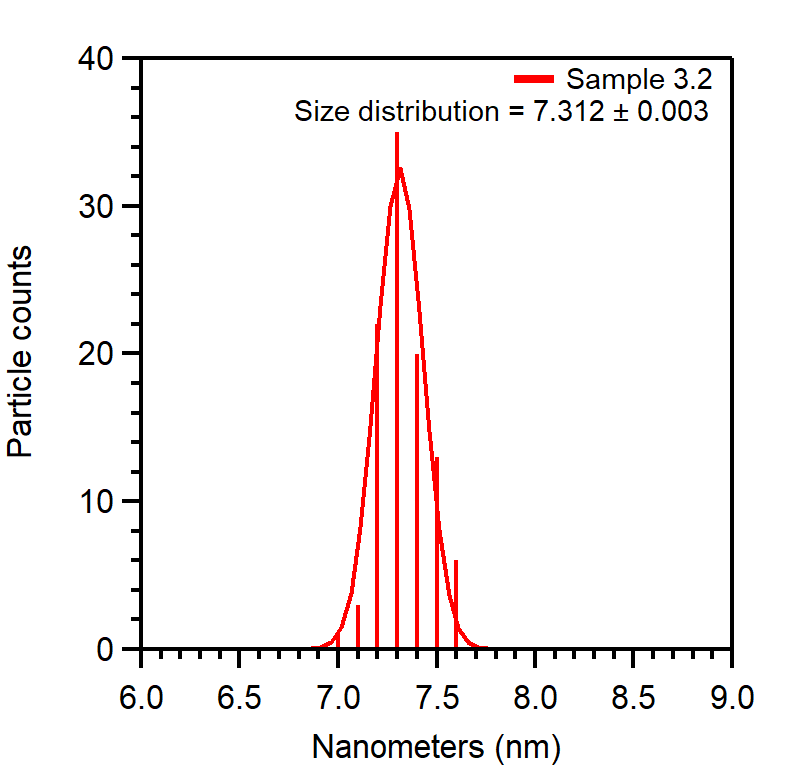

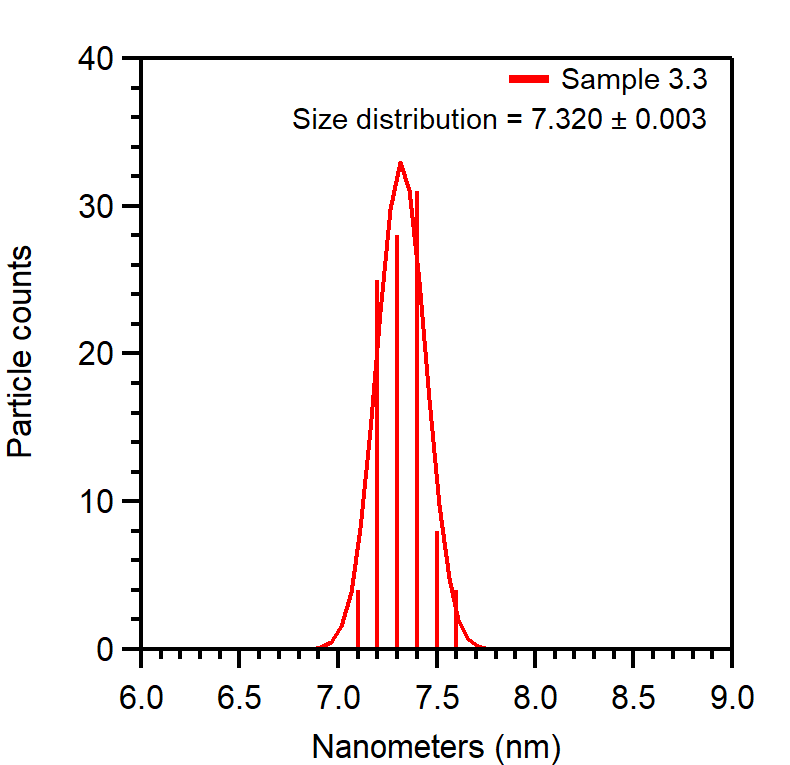
**

**Figure S3-3:** Size distribution of PbS/CdS core/shell series 3: a. PbS core 3, b. Sample 3.1, c. Sample 3.2, and d. Sample 3.3.

**S4: Cation exchange reaction kinetics**

**
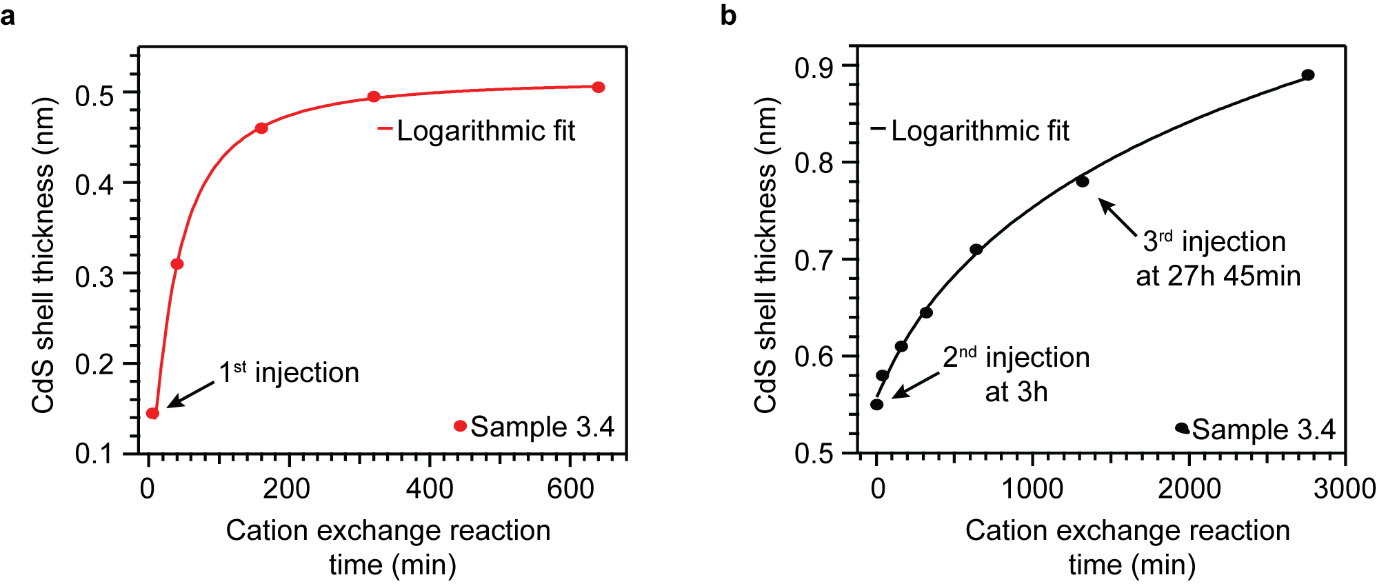
**

**Figure S4:** Kinetics of cation exchange reaction of Sample 3.4: a. 1^st^ injection of Cd-oleate, reaching a plateau until the formation of ⁓0.5 nm CdS shell and b. 2^nd^ and 3^rd^ injection of Cd-oleate at 3h and at 27h 45min of reaction, respectively. In both cases shell growth slows down logarithmically.

**S5: Photoluminescence (PL)**

**PbS/CdS core/shell series 1**

**a b**

**
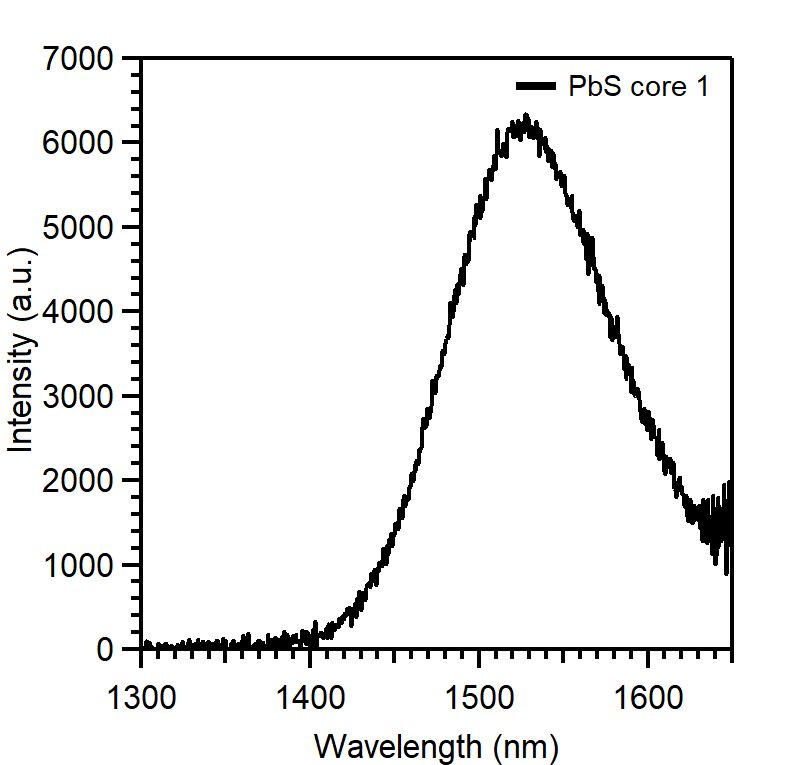

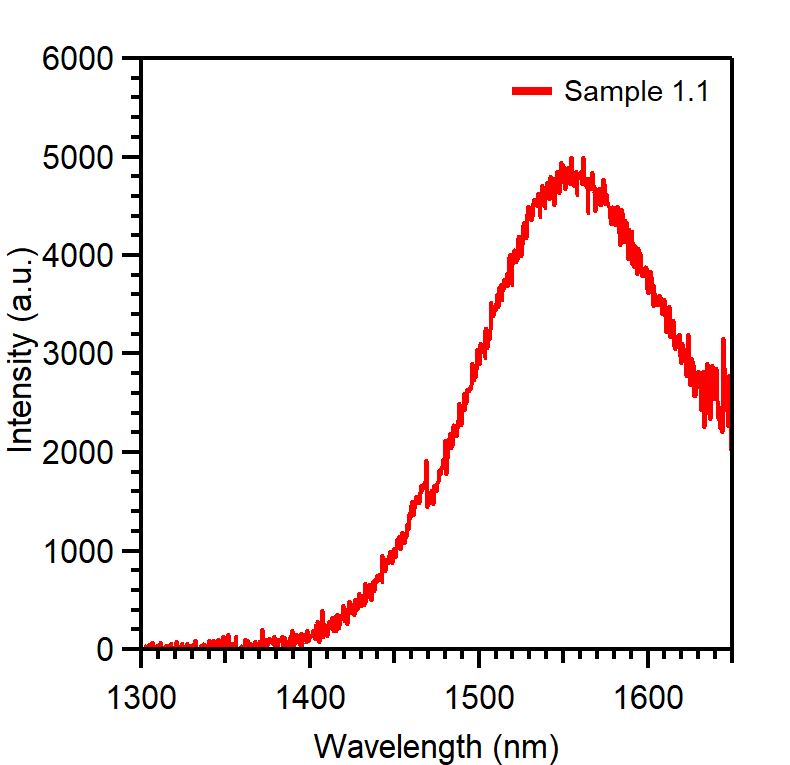
**

**c d**

**
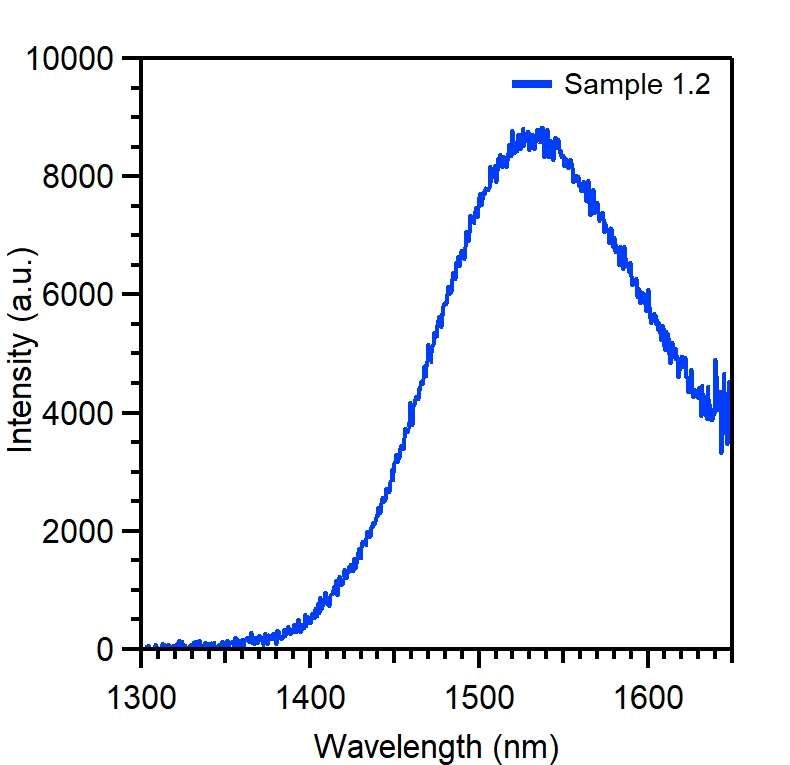

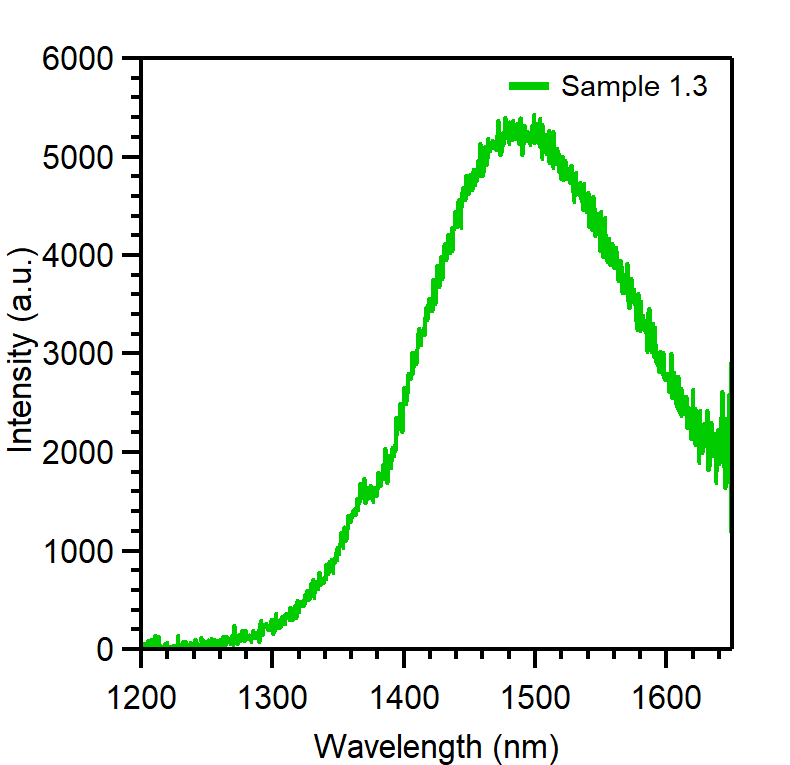
**

**Figure S5-1:** PL spectra of PbS/CdS core/shell series 1: a. PbS core 1, b. Sample 1.1, c. Sample 1.2, and d. Sample 1.3.

**PbS/CdS core/shell series 2**

**a b**

**
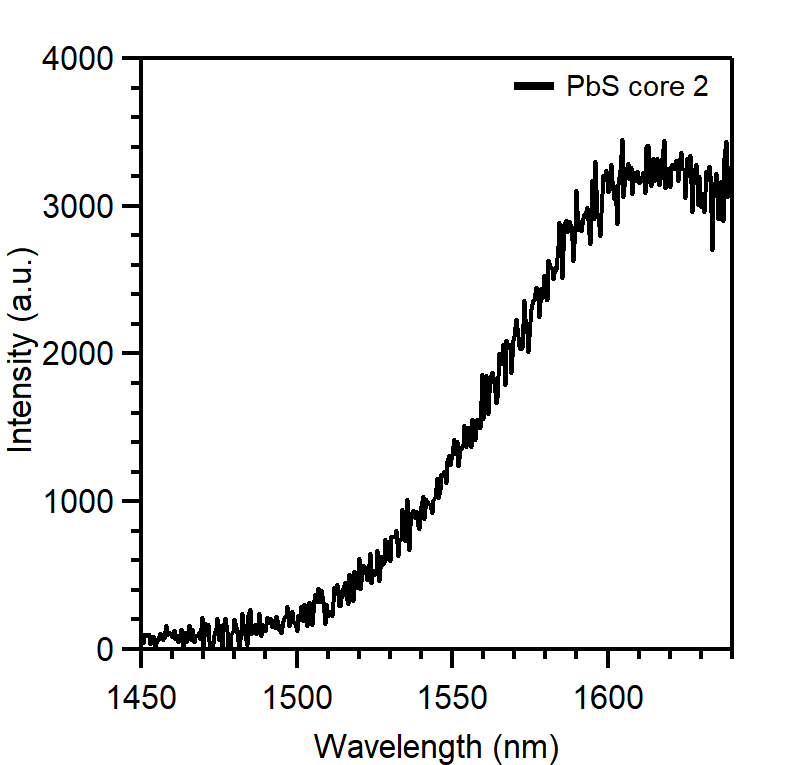

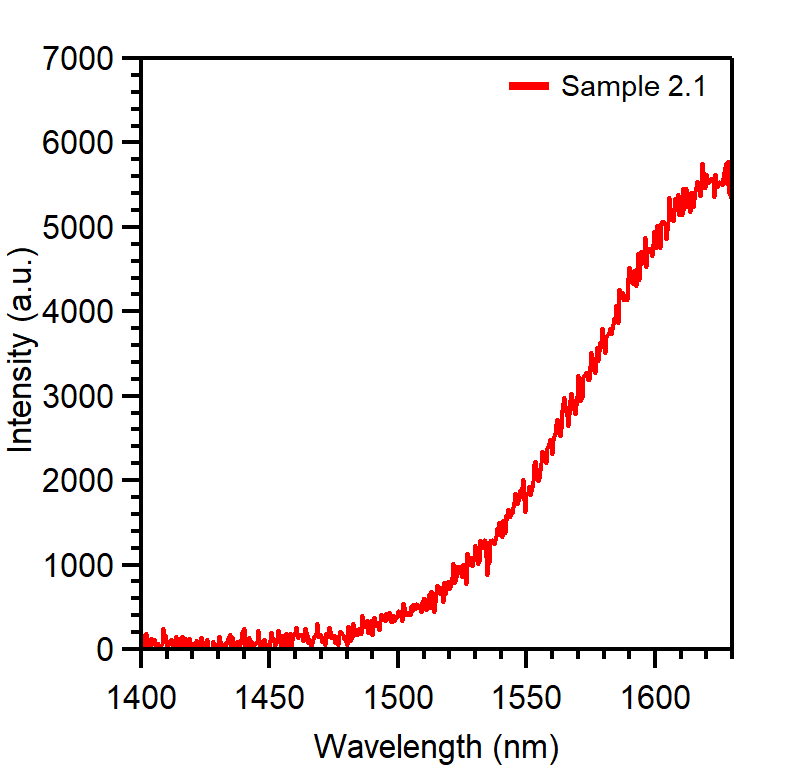
**

**c d**

**
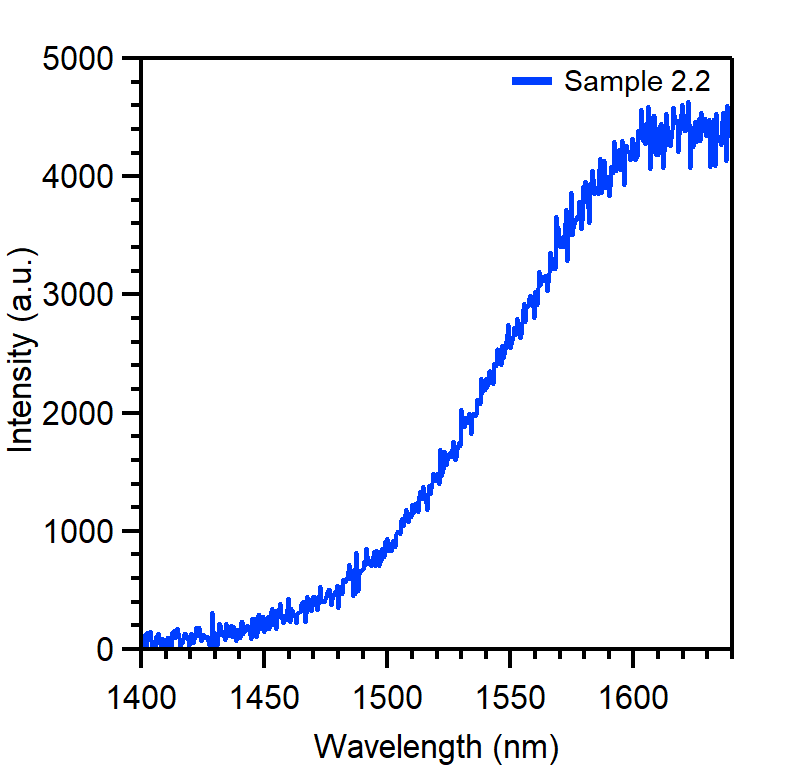

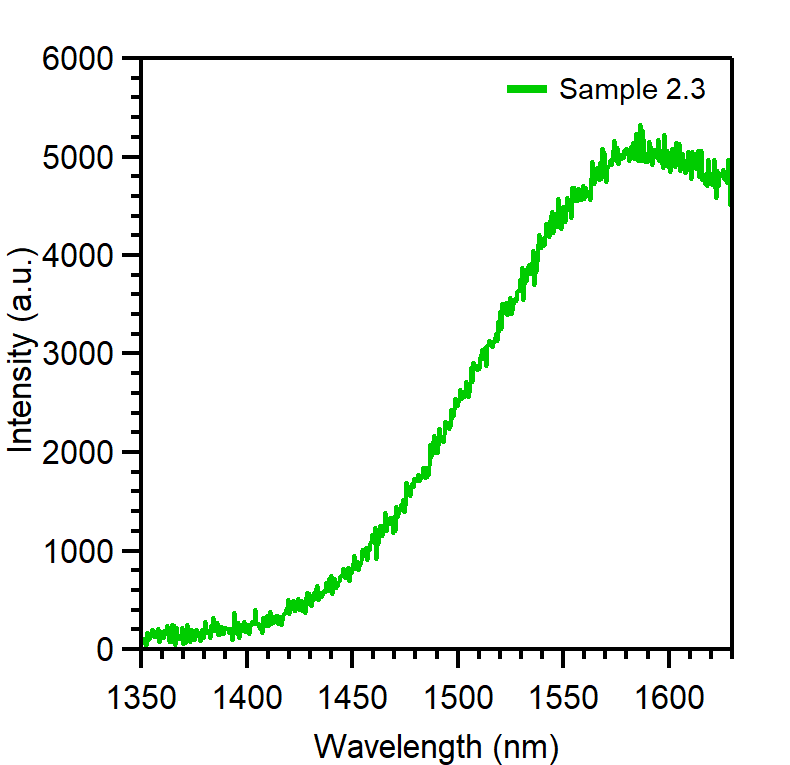
**

**Figure S5-2:** PL spectra of PbS/CdS core/shell series 2: a. PbS core 2, b. Sample 2.1, c. Sample 2.2, and d. Sample 2.3.

**PbS/CdS core/shell series 3**

**a b**

**
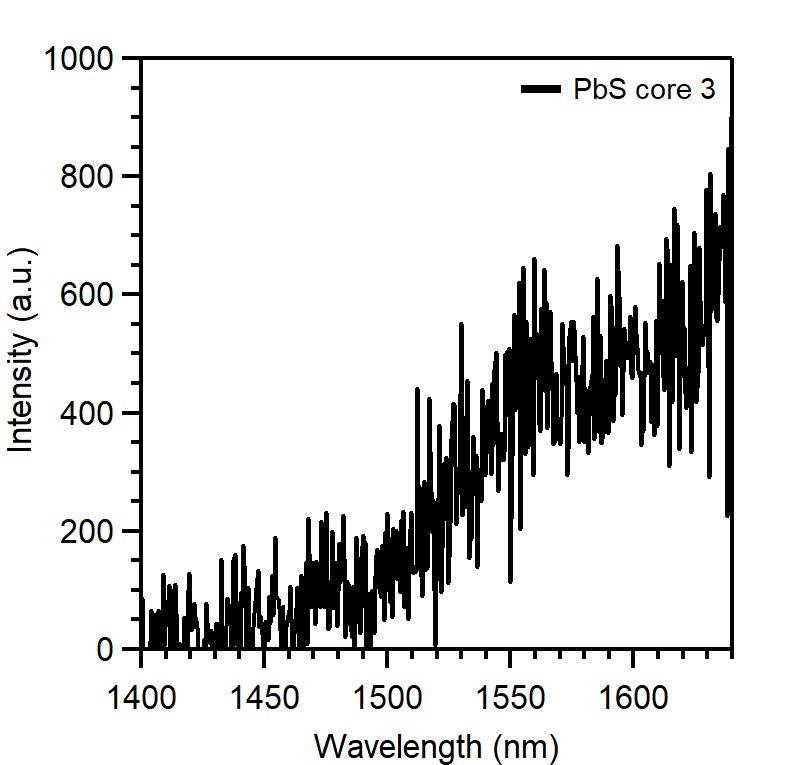

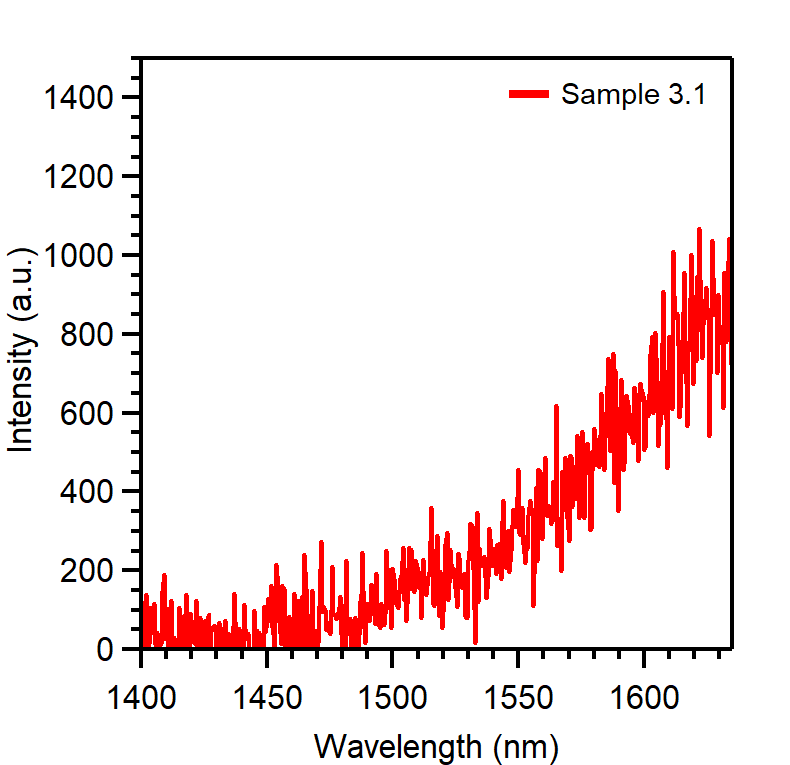
**

**c d**

**
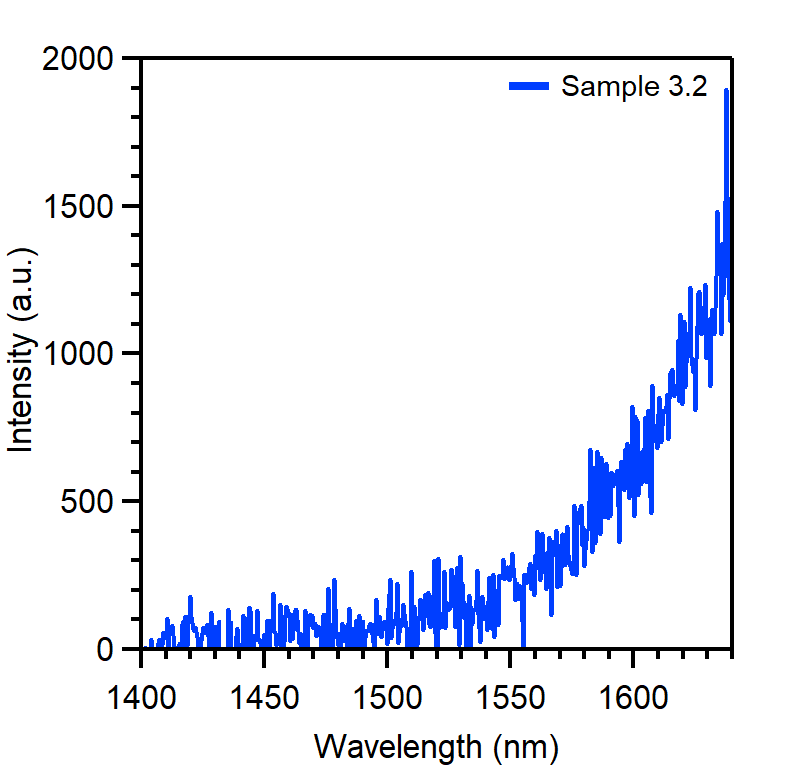

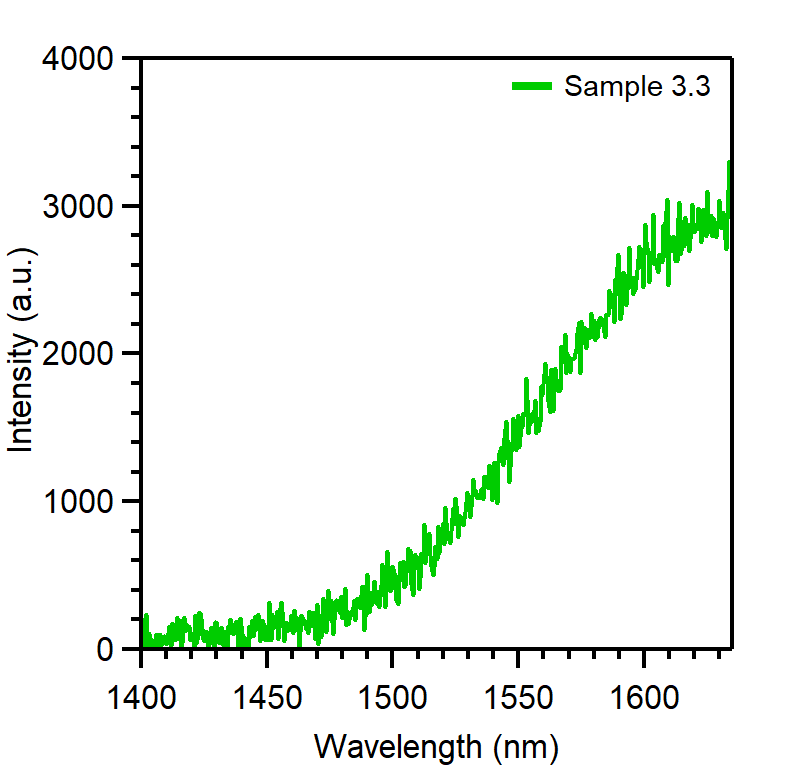
**

**e**

**
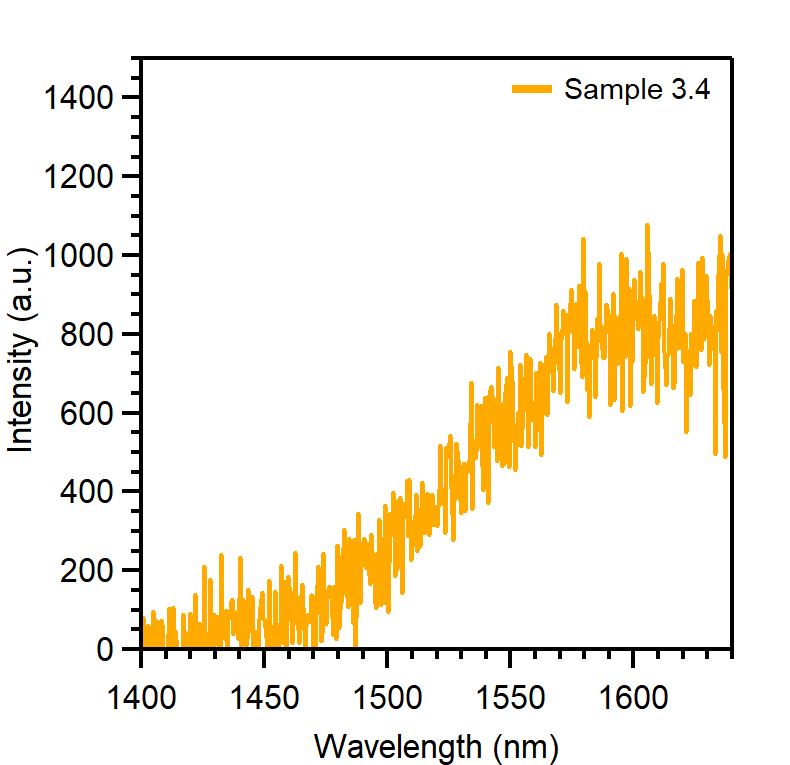
**

**Figure S5-3:** PL spectra of PbS/CdS core/shell series 3: a. PbS core 3, b. Sample 3.1, c. Sample 3.2, d. Sample 3.3, and e. Sample 3.4.

**S6: Calculations for exciton occupancy per dot <N> and <N>_gain_ threshold values**

The exciton occupancy per dot <N> was calculated based on the below:

$$<N>= \frac{E_{pulse}}{E_{CQD}^{<N> =1}}$$

where $E_{pulse}$ is the incident energy used and $E_{CQD}^{<N> =1}$ is the incident energy required for N =1 in all the excited CQD population.

$$E_{CQD}^{<N> =1}=N_{photons}^{<N> =1}\times E_{photons}^{800}$$

where $N_{photons}^{<N> =1}$ is the total number of photons needed to generate a carrier per CQD whereas $E_{photons}^{800}$ is the photon energy at the excitation wavelength 800 nm.

$$E_{photons}^{800}=\frac{hc}{\lambda}=2.483\times{10}^{-19} J$$

$$N_{photons}^{<N> =1}=\frac{N_{CQD}}{1-T-R}$$

where $N_{CQD}$ is the total excited CQD population and $(1-T-R)$ equals to the absorption of the population calculated by measuring the transmission $\left( T \right)$ and the reflection $\left( R \right)$ spectra of the film at 800 nm.

$$N_{CQD}=\frac{A_{exc}H_{film}0.92}{V_{CQD}}$$

where $A_{exc}$ is the excitation area on the film, $H_{film}$ is the thickness of each CQD film which was measured using profilometry, 0.92 is the packing density φ_L_max^4^ of a close pack film of cuboctahedra shaped CQDs and $V_{CQD}$ is the volume of the quantum dots.

$$A_{exc}=\pi R_{beam}^{2}$$

where $R_{beam}$ is the radius of the laser beam equal to 0.75 mm.

$$V_{CQD}=\frac{4\pi R_{CQD}^{3}}{3}$$

where $R_{CQD}$ is the radius of each quantum dot.

<N>_gain_ threshold values were extracted for each PbS/CdS CQD sample at the condition

-Δα/α_PbS/CdS_ = 1, corresponding to the onset of the stimulated-emission regime.

**S7: Time-Resolved Photoluminescence (TRPL)**


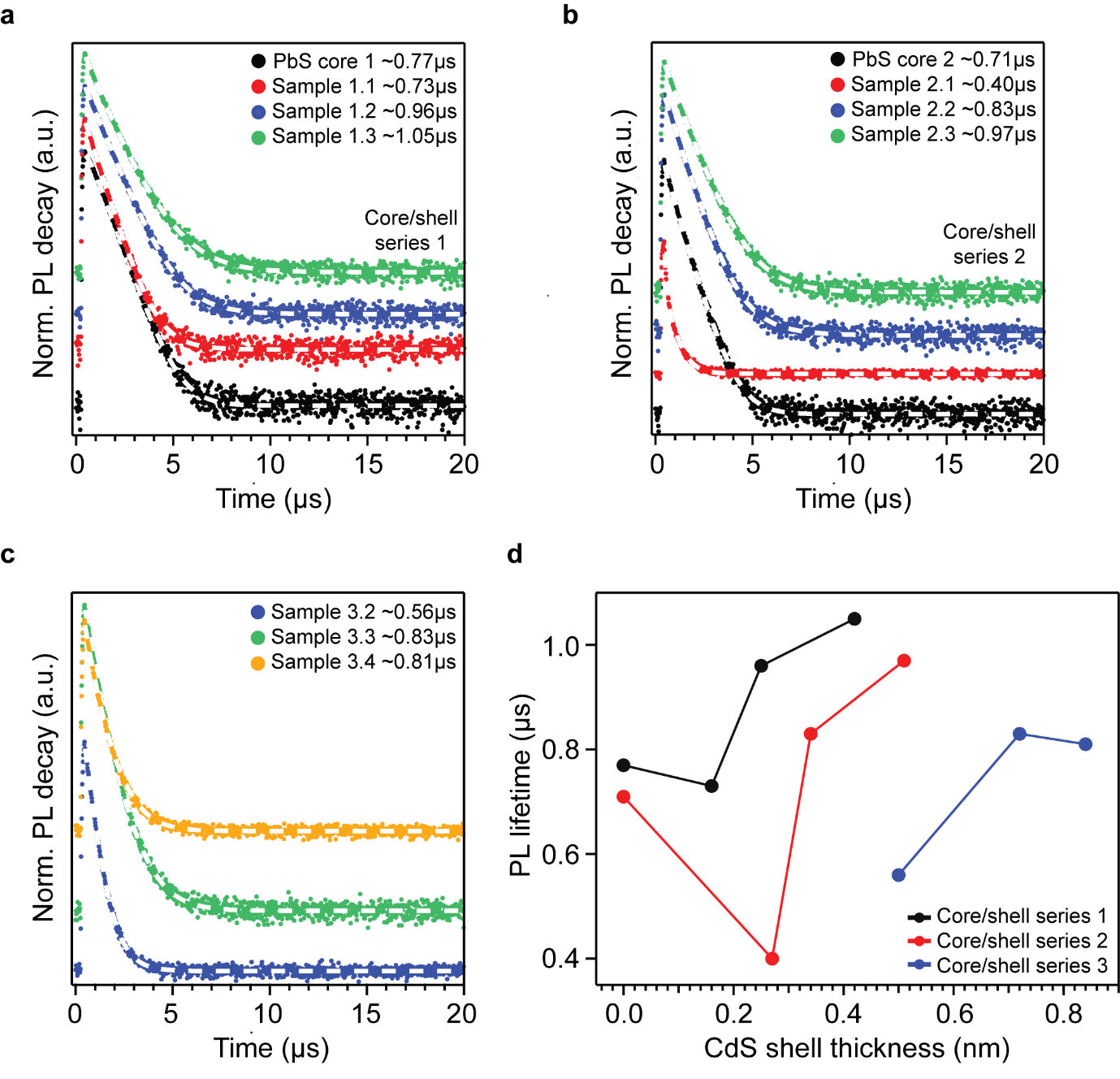


**Figure S7:** TRPL spectra of PbS/CdS core/shell series: a. Core/shell series 1, b. Core/shell series 2, c. Core/shell series 3 and d. Observed lifetimes in relation with CdS shell thickness.

**S8: Examination of possible electron doping induced by ligand exchange**

**
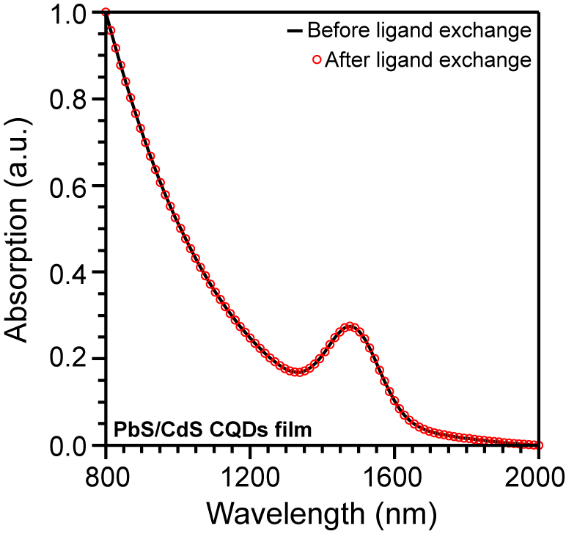
**

**Figure S8:** UV–NIR absorption spectra of a solid-state PbS/CdS CQDs film before and after ligand exchange using an EMII/MPA solution in methanol. The spectra are normalized at high energy (800 nm) and show no quenching of the band-edge excitonic absorption after ligand exchange, indicating no evidence of electron doping in the film.

**S9: Photo-stability measurements**

**
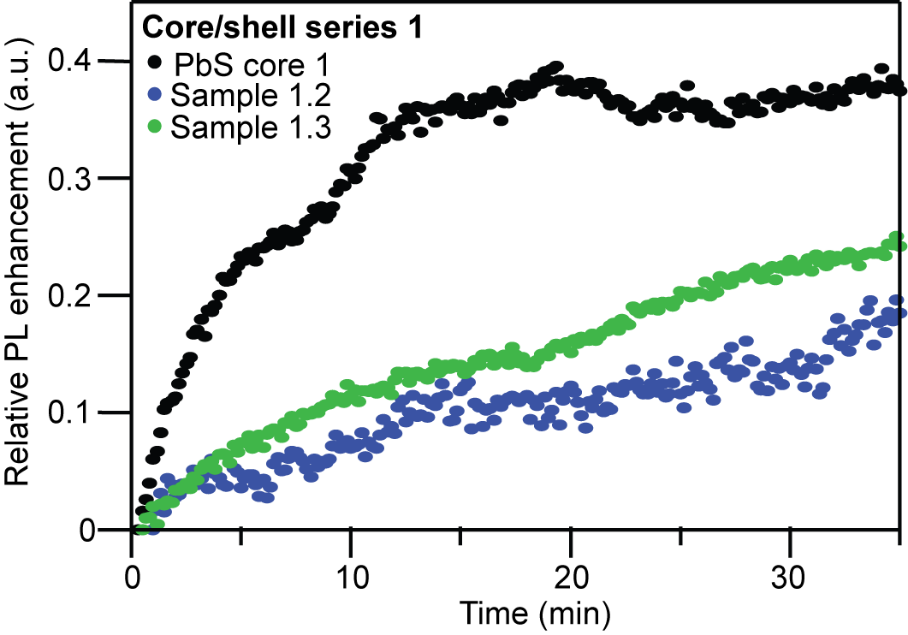
**

**Figure S9:** Relative PL enhancement of PbS core 1, Sample 1.2 (intermediate shell) and Sample 1.3 (thick shell) under 1 W/mm^2^ excitation for over 30 minutes.

**S10: Correlated TEM images from photo-stability measurements**

**PbS/CdS core/shell series 1**

**
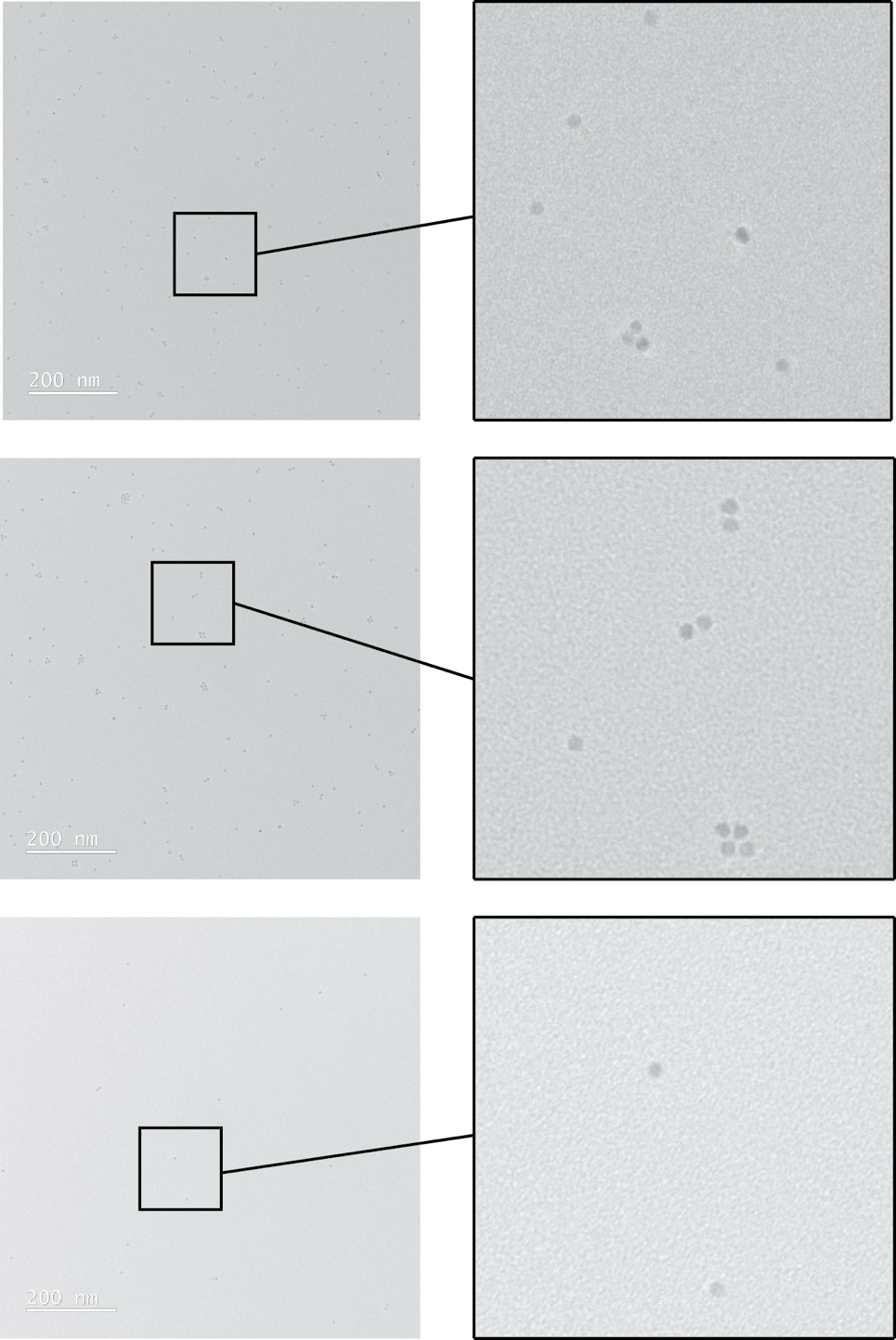
**

c

b

a

**Figure S10:** TEM images of PbS/CdS core/shell series 1 at ultra-high dilution: a. PbS core 1, b. Sample 1.2 (intermediate shell) and c. Sample 1.3 (thick shell). At this dilution CQDs are present as single CQDs, dimers and trimers, as well as a few higher-number clusters (5-6 CQDs). There is no evidence of CQD stacking.

**S11: FT-IR spectra of PbS and PbS/CdS CQDs films**

**
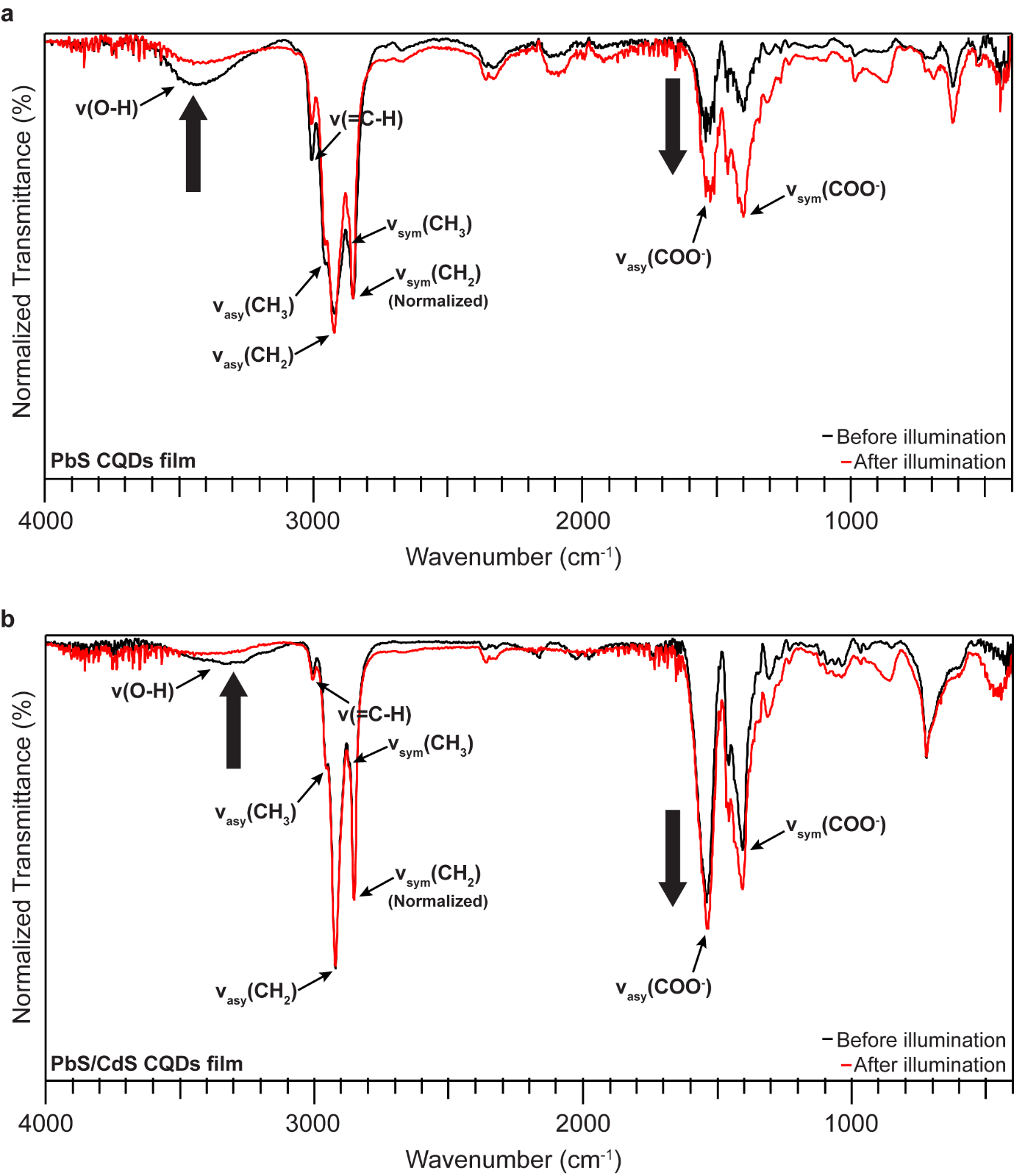
**

**Figure S11:** FT-IR spectra of PbS and PbS/CdS CQDs films before and after illumination for 30 minutes (Normalized at v_sym_(CH_2_) vibration mode – Black arrows showing the increase of COO⁻ modes and the decrease of O-H mode, respectively after illumination).

**Table S11.** Closer analysis of FT-IR patterns of PbS and PbS/CdS CQDs films before and after illumination for 30 minutes. Wavenumber values of the peak positions corresponding to the stretching vibrations of the characteristic functional groups of oleic acid.^5–9^

| **Stretching vibrations** | **PbS CQDs film** | | | | **PbS/CdS CQDs film** | | |
| --- | --- | --- | --- | --- | --- | --- | --- |
|  | **Before**  **illum. (cm^-1^)** | | **After**  **illum. (cm^-1^)** | **Before**  **illum. (cm^-1^)** | | | **After**  **illum. (cm^-1^)** |
| v(O-H) | ⁓3400 | ⁓3400 | | | ⁓3300 | ⁓3300 | |
| v(=C-H) | 3005 | 3005 | | | 3005 | 3005 | |
| v_asy_(CH_3_) | 2955 | 2955 | | | 2954 | 2954 | |
| v_asy_(CH_2_) | 2921 | 2923 | | | 2921 | 2922 | |
| v_sym_(CH_3_) | 2872 | 2872 | | | 2872 | 2872 | |
| v_sym_(CH_2_) | 2853 | 2853 | | | 2852 | 2852 | |
| v_asy_(COO⁻) | 1540 | 1540 | | | 1541 | 1541 | |
| v_sym_(COO⁻) | 1399 | 1399 | | | 1405 | 1407 | |

**References**

(1) Galindo, P. L.; Kret, S.; Sanchez, A. M.; Laval, J.-Y.; Yáñez, A.; Pizarro, J.; Guerrero, E.; Ben, T.; Molina, S. I. The Peak Pairs Algorithm for Strain Mapping from HRTEM Images. *Ultramicroscopy* **2007**, *107* (12), 1186–1193. https://doi.org/10.1016/j.ultramic.2007.01.019.

(2) Kilaas, R. Optimal and Near‐optimal Filters in High‐resolution Electron Microscopy. *Journal of Microscopy* **1998**, *190* (1–2), 45–51. https://doi.org/10.1046/j.1365-2818.1998.3070861.x.

(3) Peña, F. de la; Prestat, E.; Fauske, V. T.; Burdet, P.; Lähnemann, J.; Jokubauskas, P.; Furnival, T.; Nord, M.; Ostasevicius, T.; MacArthur, K. E.; Johnstone, D. N.; Sarahan, M.; Taillon, J.; Aarholt, T.; pquinn-dls; Migunov, V.; Eljarrat, A.; Caron, J.; Francis, C.; Nemoto, T.; Poon, T.; Mazzucco, S.; actions-user; Tappy, N.; Cautaerts, N.; Somnath, S.; Slater, T.; Walls, M.; Winkler, F.; Ånes, H. W. Hyperspy/Hyperspy: Release v1.7.3, 2022. https://doi.org/10.5281/zenodo.7263263.

(4) Torquato, S.; Jiao, Y. Dense Packings of Polyhedra: Platonic and Archimedean Solids. *Phys. Rev. E* **2009**, *80* (4). https://doi.org/10.1103/physreve.80.041104.

(5) Deacon, G. B.; Phillips, R. J. Relationships between the Carbon-Oxygen Stretching Frequencies of Carboxylato Complexes and the Type of Carboxylate Coordination. *Coordination Chemistry Reviews* **1980**, *33* (3), 227–250. https://doi.org/10.1016/S0010-8545(00)80455-5.

(6) Cass, L. C.; Malicki, M.; Weiss, E. A. The Chemical Environments of Oleate Species within Samples of Oleate-Coated PbS Quantum Dots. *Anal. Chem.* **2013**, *85* (14), 6974–6979. https://doi.org/10.1021/ac401623a.

(7) Kennehan, E. R.; Munson, K. T.; Doucette, G. S.; Marshall, A. R.; Beard, M. C.; Asbury, J. B. Dynamic Ligand Surface Chemistry of Excited PbS Quantum Dots. *J. Phys. Chem. Lett.* **2020**, *11* (6), 2291–2297. https://doi.org/10.1021/acs.jpclett.0c00539.

(8) Sowa, J. K.; Roberts, S. T.; Rossky, P. J. Exploring Configurations of Nanocrystal Ligands Using Machine-Learned Force Fields. *J. Phys. Chem. Lett.* **2023**, *14* (32), 7215–7222. https://doi.org/10.1021/acs.jpclett.3c01618.

(9) Zhang, J.; Zhang, H.; Cao, W.; Pang, Z.; Li, J.; Shu, Y.; Zhu, C.; Kong, X.; Wang, L.; Peng, X. Identification of Facet-Dependent Coordination Structures of Carboxylate Ligands on CdSe Nanocrystals. *J. Am. Chem. Soc.* **2019**, *141* (39), 15675–15683. https://doi.org/10.1021/jacs.9b07836.
